# IL-1β priming triggers an adaptive stress response that enhances pancreatic β-cell resilience to subsequent cytotoxic inflammatory insult

**DOI:** 10.1038/s41419-025-08059-0

**Published:** 2025-10-21

**Authors:** Carolina Sétula, Ingrid Pensado-Evans, Andrea Scelza-Figueredo, Miranda Sol Orellano, Ignacio Rodríguez-Valero, Eduardo Spinedi, Raghavendra G. Mirmira, Luz Andreone, Marcelo Javier Perone

**Affiliations:** 1 Immuno-Endocrinology, Diabetes & Metabolism Laboratory, Instituto de Investigaciones en Medicina Traslacional (IIMT), CONICET-Universidad Austral, Pilar, Argentina; 2 Facultad de Ciencias Biomédicas, Universidad Austral, Pilar, Argentina; 3 Centre of Experimental and Applied Endocrinology (CENEXA, UNLP-CONICET), La Plata Medical School, Universidad Nacional de La Plata, La Plata, Argentina; 4 Department of Medicine and the Kovler Diabetes Center, The University of Chicago, Chicago, Illinois USA

**Keywords:** Cell death, Endocrine system and metabolic diseases

## Abstract

Pancreatic β-cells fine-tune glucose homeostasis through insulin secretion. The endoplasmic reticulum (ER) is critical for insulin production, relying on the unfolded protein response (UPR) to adapt to the body’s fluctuating demands. Islets from both type 1 (T1D) and type 2 diabetes (T2D) exhibit inflammation, β-cell dysfunction, and loss. ER stress is present in the inflamed islets of autoimmune diabetes-prone mice and individuals with T1D and T2D. Inflammatory cytokines induce ER stress and disrupt UPR regulation, driving β-cell apoptosis and contributing to diabetes development. Inflammatory cytokines, *e.g*., IL-1β, impair β-cell function and survival, contributing to diabetes pathogenesis by inducing stress, altering gene expression, driving dedifferentiation, and reducing insulin production. Paradoxically, β-cells exhibit a high density of IL-1R1, and IL-1R1/KO mice display impaired glucose tolerance and reduced insulin secretion. Postprandial IL-1β secreted by macrophages helps maintain blood glucose homeostasis. These observations suggest that circulating low IL-1β concentrations may have physiologically relevant roles; however, their effects on β-cell function and survival remain unclear due to conflicting reports. Preconditioning β-cells with physiological circulating levels of IL-1β (IL-1β^low^) induced a resilient state, protecting them from pro-inflammatory cytokine (CYT)-induced cell death while preserving glucose-stimulated insulin secretion through hormesis. IL-1β^low^-treated INS-1E cells reduced CYT-induced NO secretion by suppressing NF-κB signaling and decreasing iNOS expression, correlating with reduced β-cell death. IL-1β^low^ conditioning reduced ER stress and upregulated p-eIF2α in response to CYT, thereby enhancing the expression of ER chaperones and biomarkers linked to improved β-cell identity/functionality. Transcriptomic analysis revealed that IL-1β^low^ preconditioning mitigated the CYT-induced loss of genes involved in β-cell function/identity, and suppressed the expression of genes linked to NF-κB signaling, cytokine-induced inflammation, and apoptosis. IL-1β^low^ treatment counteracted the upregulation of stress-related genes triggered by pro-inflammatory stimuli. Enhancing IL-1β^low^-induced stress-response hormesis may provide a novel strategy to sustain β-cell function and survival during harmful diabetic inflammation.

## Introduction

Pancreatic β-cells play a crucial role in maintaining glucose homeostasis by secreting insulin. Type 1 (T1D) and type 2 diabetes (T2D), the most common clinical presentations of diabetes, are both characterized by β-cell dysfunction and loss.

The endoplasmic reticulum (ER) in β-cells is essential for protein folding and insulin synthesis, with the unfolded protein response (UPR) helping to manage fluctuating insulin production demands [1]. ER stress markers are present in the inflamed islets of autoimmune diabetes-prone nonobese diabetic mice [2] and individuals with T1D [3], and T2D [4].

Hormesis is a phenomenon in which a cytotoxic agent, in small doses, benefits organisms. Cells exposed to low toxin levels can resist subsequent high-dose exposures [5]. It is suggested that the hormetic response to lifestyle detrimental factors such as poor diet, sedentarism, and stress may influence protection levels and impact T2D progression [6].

Inflammatory cytokines, such as IL-1β, TNF-α and IFN-γ negatively affect β-cell function and survival, contributing to the pathogenesis of both T1D and T2D [7–10]. Inflammation exacerbates ER stress and activates the UPR, which, when prolonged or dysregulated, leads to β-cell apoptosis [11–14]. Therefore, restoring ER homeostasis in β-cells has been proposed as a potential strategy to alleviate T1D [15]. Pro-inflammatory cytokines, particularly IL-1β, drive β-cell dedifferentiation by inducing cellular stress, altering gene expression, and reducing insulin production. This involves the downregulation of key transcription factors essential for β-cell identity (Pdx-1, Mafa, FoxO1, Nkx6.1). IL-1β and other inflammatory cytokines induce the expression of inducible nitric oxide synthase (iNOS) in β-cells, leading to nitric oxide (NO) accumulation. Elevated NO disrupts insulin secretion, protein synthesis, oxidative metabolism, and causes DNA damage, impairing β-cell function and health [16, 17]. IFN-γ amplifies the harmful effects of IL-1β on β-cells [18]. In contrast, acute low concentrations of IL-1β stimulate insulin release in rat islets [19], underscoring its complex and context-dependent effects on β-cell function and insulin regulation. Meanwhile, the long-term impact of very low IL-1β concentrations on β-cells remains uncertain.

In this study, we explored the role of IL-1β-mediated hormesis in defending β-cells against dysfunction and death induced by pro-inflammatory cytokines. Our findings show that IL-1β, at basal physiological concentrations, triggers a hormetic response in β-cells, enhancing their resilience to future cytotoxic cytokine challenges. Inducing hormetic responses in vivo offers a promising strategy to prevent β-cell decline in diabetes and warrants further investigation.

## Results

### Preconditioning INS-1E cells with low concentrations of IL-1β mitigates NO secretion in response to a cytotoxic pro-inflammatory cytokine challenge

**Fig. 1:**
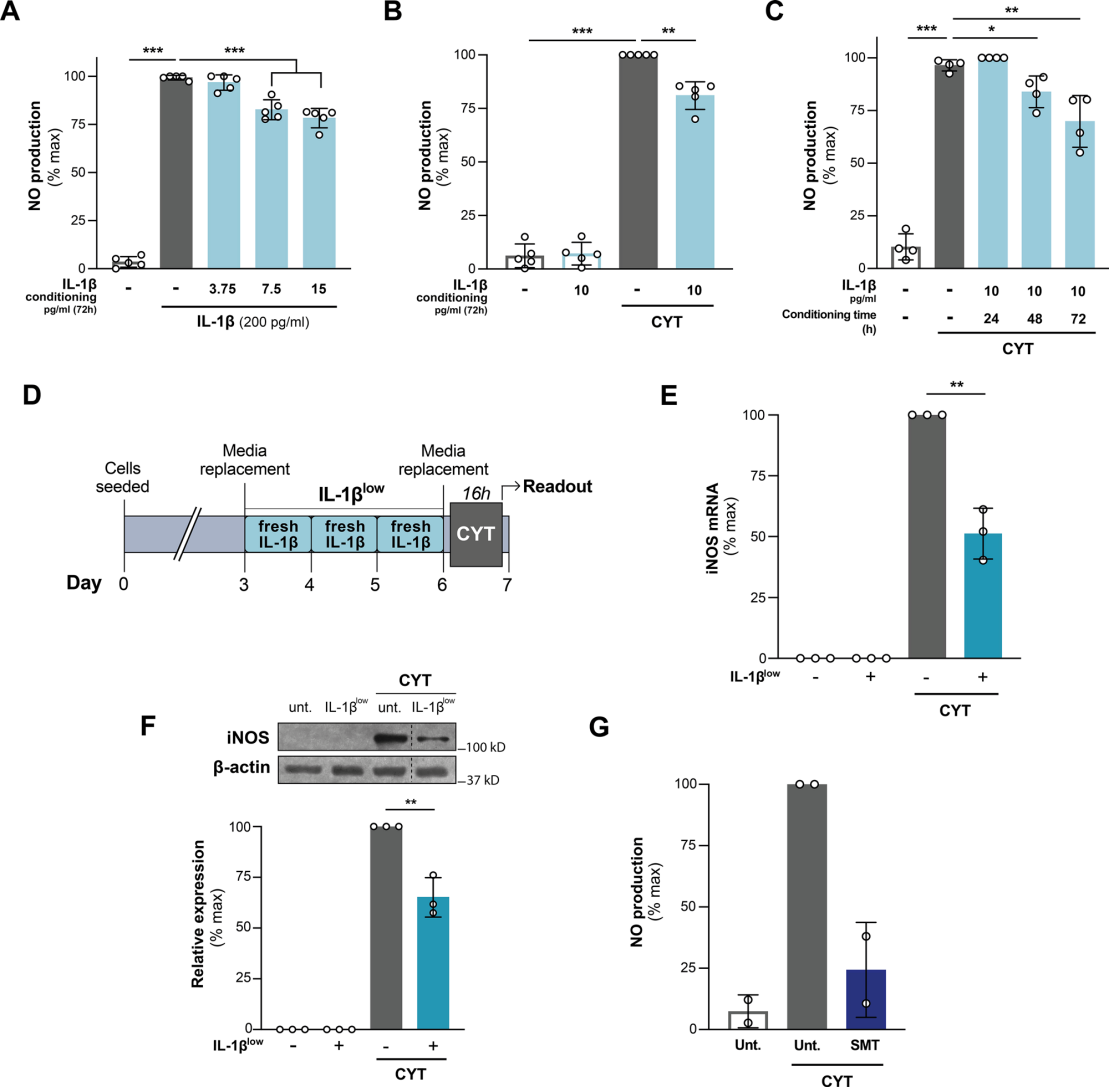
Preconditioning with low concentrations of IL-1β reduces pro-inflammatory cytokine-induced iNOS expression and activity in INS-1E cells. **A**, **B** INS-1E cells were treated for 72 h with IL-1β (3.75, 7.5, 15 pg/ml or 10 pg/ml as indicated) and subsequently challenged or not with IL-1β 200 pg/ml or IL-1β 100 pg/ml + IFN-γ 5 ng/ml (CYT) for 16 h, *n* = 5. **C** INS-1E cells were conditioned with IL-1β (10 pg/ml) for 24, 48, or 72 h, as indicated, and subsequently challenged or not with CYT for 16 h, *n* = 4. **D** Schematic diagram of IL-1β preconditioning. Cells were treated with IL-1β (10 pg/ml), added fresh every 24 h for 72 h without media change (IL-1β^low^). After media renewal, cells were challenged with a proinflammatory mix (CYT: IL-1β 100 pg/ml + IFN-γ 5 ng/ml). **E**, **F** INS-1E cells were treated with IL-1β^low^ and subsequently challenged or not with CYT for 6 h, *iNOS* mRNA and protein expression were analyzed by RT-qPCR (mRNA levels normalized to HPRT, *n* = 3) and Western blot (β-actin was used as loading control, *n* = 3), respectively. **G** NO secretion induced by CYT in the presence of 5-methylisothiourea sulfate (SMT), a selective iNOS inhibitor, was assessed in two independent experiments. NO levels in the conditioned media were assessed by Griess reaction and normalized to total cell protein content. Data are shown as mean ± SD. (*) *p* < 0.05, (**) *p* < 0.01, (***) *p* < 0.001.

Increases in iNOS-derived NO act as a trigger for pro-inflammatory cytokine-mediated ER stress and death in a β-cell-specific manner [20]. INS-1E cells exposed to IL-1β (200 pg/ml) for 16 h secreted significant amounts of NO (228 ± 33.9 pmol/μg protein) into the culture medium. Notably, priming these cells with IL-1β (7.5 and 15 pg/ml for 72 h) reduced NO secretion in response to IL-1β (200 pg/ml/16 h) (Fig. 1A). In a similar experiment, IL-1β preconditioning reduced NO secretion (Supplementary Figure 1A) in INS-1E cells compromised by a 16 h exposure to a cytokine mixture containing IL-1β (200 pg/ml) and TNF-α (8 ng/ml).

TNF-α and IL-1β, together with IFN-γ trigger similar signaling pathways [21]. Thus, INS-1E cells were challenged with IL-1β 100 pg/ml + IFN-γ 5 ng/ml (CYT). IL-1β preconditioning reduced CYT-induced NO secretion (Fig. 1B), with effects starting at 48 h and persisting through 72 h (Fig. 1C). Hereafter, IL-1β (10 pg/ml for 72 h) is referred to as IL-1β^low^. A schematic diagram of the treatment protocol is shown in Fig. 1D.

To explore the mechanisms behind reduced NO secretion in IL-1β^low^-preconditioned INS-1E cells, we examined iNOS expression and found decreased mRNA (*p* < 0.01; Fig. 1E) and protein levels (*p* < 0.01; Fig. 1F) following CYT-challenge. The iNOS-specific inhibitor SMT abolished NO secretion, confirming CYT acts via iNOS in INS-1E cells (Fig. 1G). Unlike IL-1β^low^, IFN-γ preconditioning did not reduce CYT-induced NO secretion (Supplementary Figure 1B).

The IL-1β^low^ effect was reversible, lasting about 2 weeks after treatment, as shown by reduced CYT-induced NO secretion (Supplementary Figure 1C).

### Priming INS-1E cells with IL-1β^low^ impairs the NF-kB pathway activation triggered by the pro-inflammatory cytokine mixture

NF-κB activation links CYT-induced NO production, β-cell dysfunction, and apoptosis.

**Fig. 2:**
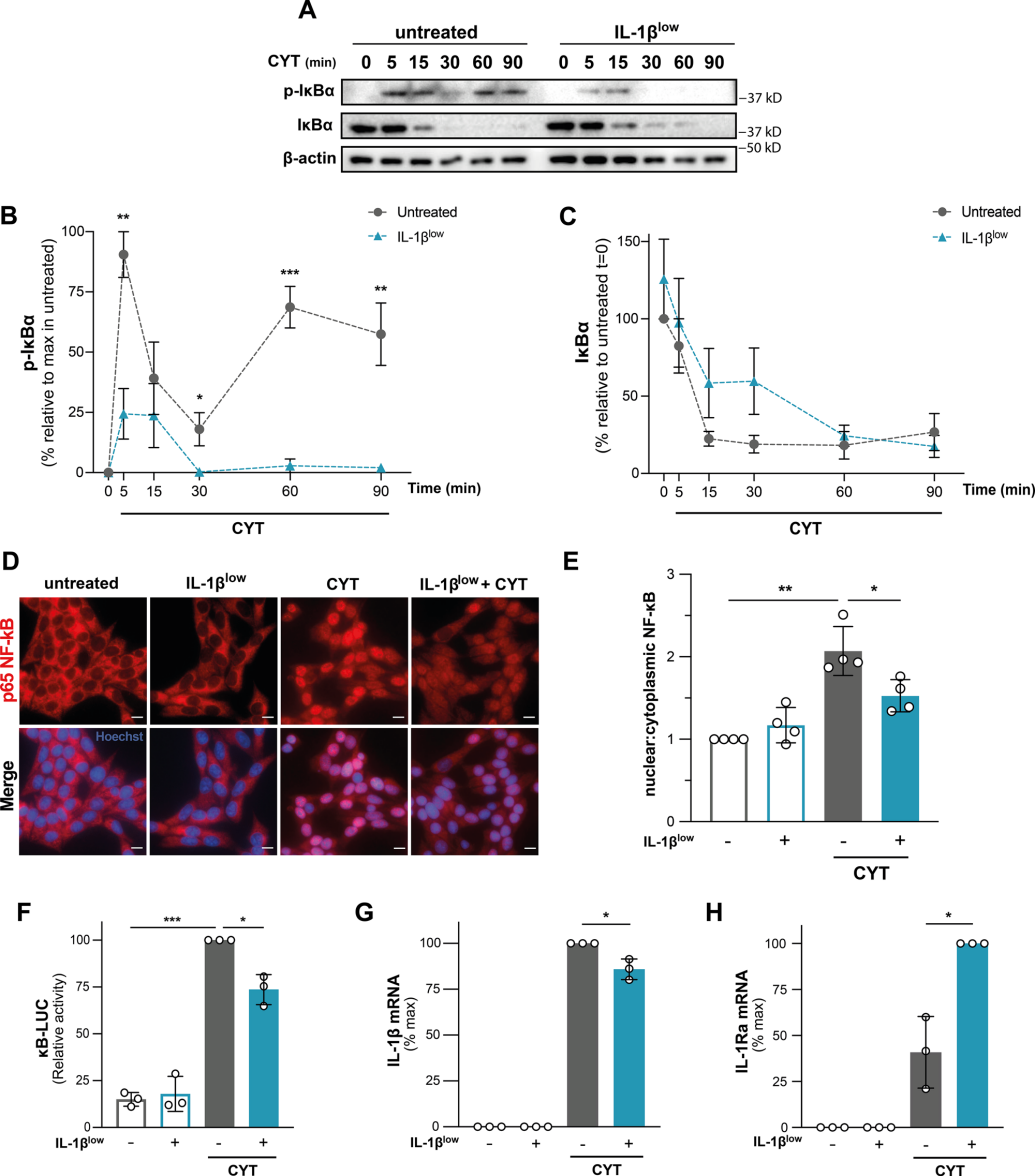
Priming with IL-1β^low^ impairs cytokine-triggered NF-κB pathway activation in INS-1E cells. **A**–**C** INS-1E cells were preconditioned with IL-1β^low^ and then challenged or not with CYT. After indicated time, protein levels of phospho-IκBα and total-IκBα were analyzed by Western blot. β-actin was used as a loading control. Data are shown as mean ± SEM, *n* = 5. **D**, **E** INS-1E cells were treated as described in (**A**), followed by 30 min stimulation with CYT. Cellular localization of p65 NF-κB immunostaining (red) was analyzed by fluorescence microscopy; nuclei were counterstained with Hoechst (blue). Scale bars: 10 μm. Nuclear-to-cytoplasmic ratio was quantified based on analysis of ten high-power fields per condition, *n* = 4. **F** IL-1β^low^-preconditioned INS-1E were transiently transfected with κB-LUC and CMV-RL reporter plasmids. After 24 h, cells were challenged or not with CYT for 16 h. Firefly luciferase (LUC) activity was then measured and normalized to Renilla luciferase (RL) activity as control for transfection efficiency, *n* = 3. **G** *IL-1β mRNA* and **H**) *IL-1Ra mRNA* levels were assessed by RT-qPCR in IL-1β^low^-preconditioned INS-1E cells, with or without CYT challenge for 6 h. Relative mRNA levels were normalized to *HPRT*, *n* = 3. Data are shown as mean ± SD. (*) *p* < 0.05, (**) *p* < 0.01, (***) *p* < 0.001.

IL-1β^low^ reduced CYT-induced IκBα phosphorylation, abolishing the second peak of p-IκBα levels triggered by the pro-inflammatory cytokine mixture (*p* < 0.01; Fig. 2A–C), and reducing NF-κB p65 nuclear translocation in INS-1E cells (*p* < 0.05, IL-1β^low^ + CYT vs. CYT; Fig. 2D, E). Additionally, preincubation of INS-1E cells with IL-1β^low^ attenuated CYT-induced NF-κB transcriptional activity, as determined by a reporter assay using a plasmid with NF-κB response elements upstream of the luciferase gene (*p* < 0.05, CYT vs. IL-1β^low^ + CYT; Fig. 2F).

IL-1β promotes its own synthesis, partly via NF-κB [22, 23]. Its autocrine or paracrine production by β-cells may contribute to their damage [24]. IL-1β^low^ attenuated CYT-induced IL-1β mRNA levels (*p* < 0.05, Fig. 2G) while upregulating the transcript expression of IL-1Ra (*p* < 0.05, Fig. 2H), an endogenous IL-1β antagonist. Similar results were obtained in isolated mouse islets under IL-1β^low^ regimen and iCYT (IL-1β 100 pg/ml + IFN-γ 5 ng/ml + TNF-α 8 ng/ml) challenge (Supplementary Figure 2A, B). The increase in *IL-1Ra* and the decrease in *IL-1β* mRNA levels induced by IL-1β^low^ likely play a role in mitigating CYT-induced adverse effects on β-cells. *IL1-R1* and *IL1-R2* mRNA expression levels showed an increasing trend in INS-1E cells pretreated with IL-1β^low^ before CYT challenge (Supplementary Figure 2C, D). IL1-R2, a decoy receptor for IL-1β, may reduce signal transduction by increasing expression in response to its cognate ligand [25].

### IL-1β^low^ preconditioning enhances resilience to pro-inflammatory cytokine-induced death in INS-1E cells

**Fig. 3:**
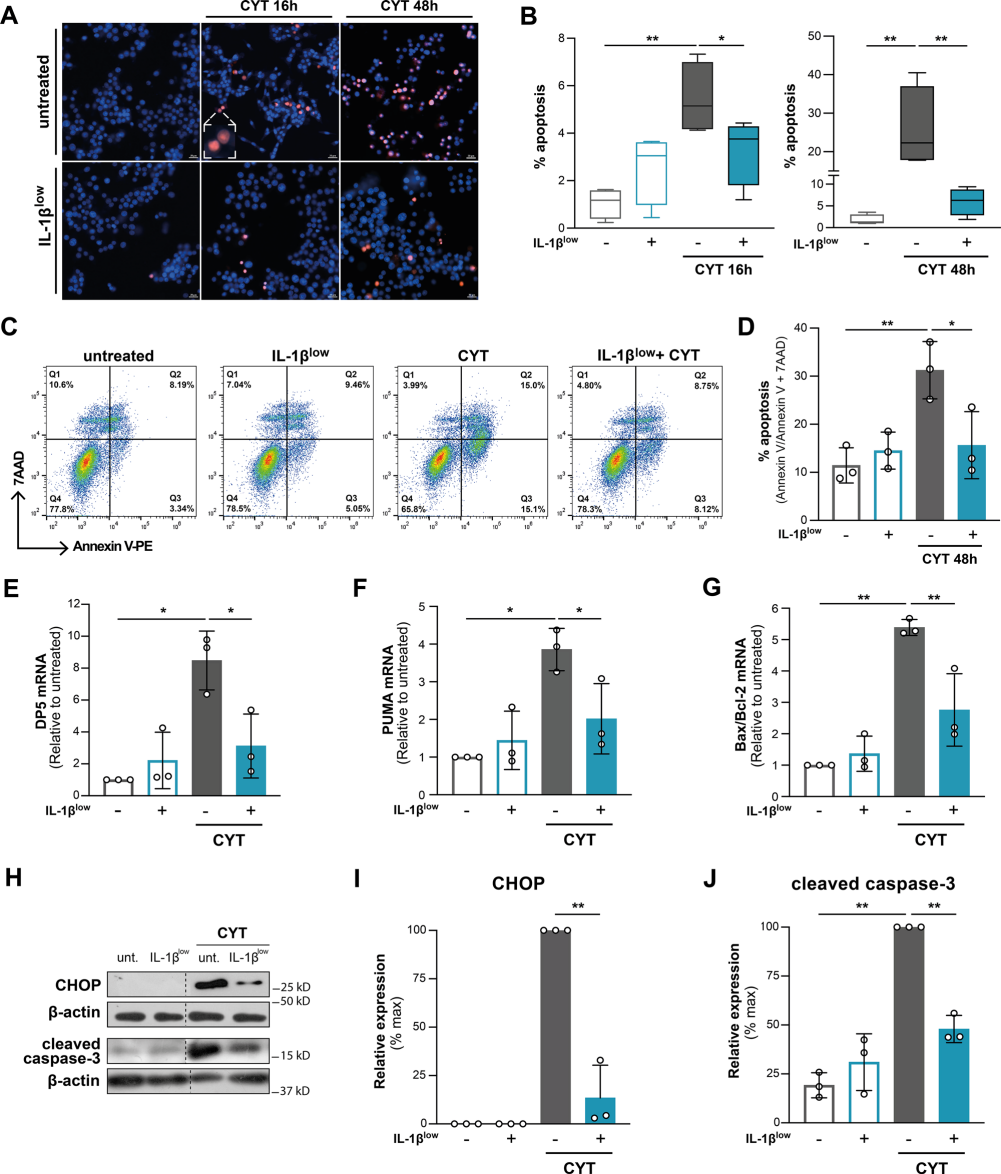
IL-1β^low^ enhances resilience to cytokine-induced death in INS-1E cells. **A**, **B** INS-1E cells were conditioned with IL-1β^low^ and subsequently challenged or not with CYT. After indicated time, cell death was analyzed by fluorescence microscopy after Hoechst (blue)/propidium iodide (red) dual staining. **A** Representative images of cells under indicated experimental conditions; scale bars 10 μm. Selected apoptotic cells are shown at higher magnification in the inset, *n* = 4. **B** Percentage of apoptotic cells based on analysis of ten high-power fields per condition, *n* = 4. **C**, **D** IL-1β^low^-preconditioned INS-1E cells were challenged or not with CYT for 48 h. Apoptosis was quantified by flow cytometry after Annexin-V/7AAD dual staining. **C** Representative dot plots of cells under each experimental condition. **D** Percentage of early (Q3) and late (Q2) apoptotic cells, *n* = 3. **E**–**J** IL-1β^low^-preconditioned INS-1E cells were treated or not with CYT for 16 h. **E** *DP5* mRNA levels, **F***PUMA* mRNA levels, and **G***Bax/Bcl-2* mRNA ratio was analyzed by RT-qPCR. Relative mRNA levels were normalized to HPRT, *n* = 3. **H**–**J** CHOP and cleaved caspase-3 protein levels were analyzed by Western blot; β-actin was used as loading control, *n* = 3. Data are shown as mean ± SD. (*) *p* < 0.05, (**) *p* < 0.01.

IL-1β^low^ reduced apoptosis/death in INS-1E cells after CYT/16 h, with protection lasting up to 48 h (*p* < 0.05 and p < 0.01 vs. CYT/16 h and CYT/48 h, respectively; Fig. 3A, B). These findings were further validated by assessing apoptosis through annexin-V/PI staining (*p* < 0.05 vs. CYT at 48 h; Fig. 3C, D).

To investigate IL-1β^low^ ‘s pro-survival mechanisms, we analyzed Bcl-2 family members involved in apoptosis. IL-1β^low^ treatment hampered the CYT-induced increases in *DP5* and *PUMA* mRNA (63.2% and 47.7%, respectively, vs. IL-1β^low^ + CYT, Fig. 3E, F) and counteracted the CYT-induced increase in *Bax/Bcl-2* mRNA ratio, suggesting reduced apoptosis susceptibility (*p* < 0.01 vs. CYT, Fig. 3G). Additionally, IL-1β^low^ reduced CYT-mediated upregulation of CHOP (86.6% vs. CYT; Fig. 3H, I) and cleaved caspase-3 (52.9% vs. CYT; Fig. 3H, J), both key mediators in the final steps of apoptosis. These results support the notion that IL-1β^low^ triggers a hormetic response, as evidenced by minimal apoptosis compared to INS-1E cells exposed to CYT alone.

### IL-1β^low^ triggers a stress-response hormesis

**Fig. 4:**
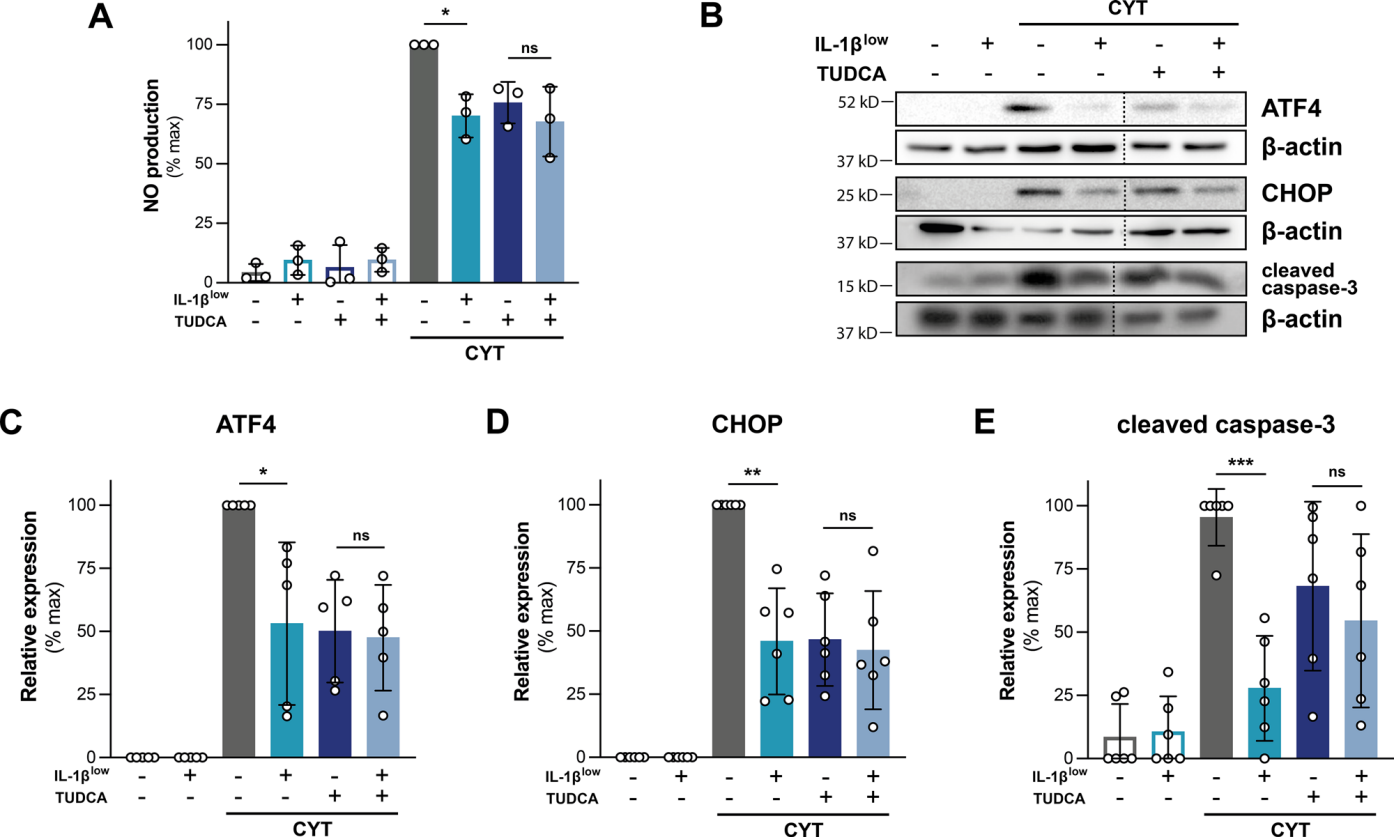
Mild ER stress is needed for an effective IL-1β^low^-induced hormesis in INS-1E cells. INS-1E cells were conditioned with IL-1β^low^ in the absence or presence of tauroursodeoxycholic acid (TUDCA, 5 μM) and subsequently challenged or not with CYT for 16 h. **A** NO levels in the culture media were quantified by the Griess reaction and normalized to total cell protein content, *n* = 3. **B–E** Protein levels of ATF4, CHOP, and cleaved caspase-3 were analyzed by Western blot. β-actin was used as a loading control, *n* = 5–6. Data are shown as mean ± SD. (*) *p* < 0.05, (**) *p* < 0.01, (***) *p* < 0.001. (ns) not significant.

We investigated if IL-1β^low^ ‘s protective effect on β-cells requires baseline ER stress for hormesis by adding TUDCA, an ER stress alleviator [15], during preconditioning. Under this condition, CYT-induced NO secretion by INS-1E cells was comparable to that observed in cells treated only with TUDCA, without IL-1β^low^ preconditioning (Fig. 4A). As expected, TUDCA reduced levels of the ER stress sensor ATF4, along with CHOP and cleaved caspase-3, which are effectors of CYT-induced ER stress-mediated cell death. Interestingly, TUDCA during the IL-1β^low^ preconditioning eliminates the stress-response hormesis, as evidenced by the unchanged expression levels of ATF4, CHOP, and cleaved caspase-3 under CYT stimulation (Fig. 4C–E). These results highlight that a minimal level of ER stress is required to elicit an effective, adaptive pro-survival stress-response hormesis in INS-1E cells against CYT insult.

### IL-1β^low^ preconditioning boosts CYT-induced eIF2α phosphorylation promoting cellular stress adaptation

**Fig. 5:**
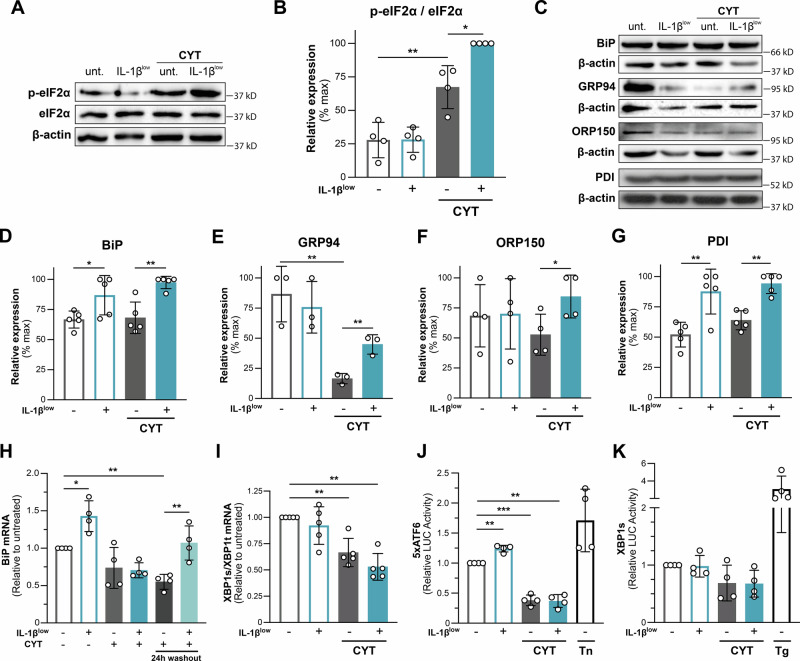
IL-1β^low^ boosts cytokine-induced eIF2α phosphorylation promoting cellular stress adaptation. INS-1E cells were preconditioned with IL-1β^low^ and subsequently challenged or not with CYT for 16 h. **A**, **B** Protein levels of phospho-eIF2α and total eIF2α were analyzed by Western blot and quantification expressed as phospho-eIF2α/total eIF2α ratio. β-actin was used as a loading control, *n* = 4. **C**–**G** Protein levels of BIP, GRP94, ORP150 and PDI were analyzed by Western blot; β-actin was used as loading control, *n* = 3-5. **H** *BIP* mRNA expression was analyzed by RT-qPCR and relative mRNA levels normalized to *HPRT*; a 24 h washout condition (without CYT) was also evaluated, *n* = 4. **I** XBP1 mRNA splicing was analyzed by RT-qPCR and expressed as *XBP1s/XBP1t* mRNA ratio. Relative mRNA levels were normalized to *HPRT*, *n* = 5. **J**, **K** IL-1β^low^-preconditioned INS-1E cells were transiently transfected with either the 5×ATF6-LUC or XBP1u-LUC reporter plasmids, along with the CMV-RL plasmid. At 24 h post-transfection, cells were challenged or not with CYT for 16 h. Firefly luciferase (LUC) activity was measured and normalized to Renilla luciferase (RL) activity, *n* = 4. Tunicamycin (2 µg/mL, Tn) and thapsigargin (50 nM, Tg) for 16 h were used as positive controls. Data are presented as mean ± SD. (*) *p* < 0.05, (**) *p* < 0.01, (***) *p* < 0.001.

In β-cells, pro-inflammatory cytokines activate the PERK branch of the UPR, leading to phosphorylation of eIF2α at Ser51 (p-eIF2α) [14, 26]. While initially protective, prolonged activation can cause β-cell dysfunction and apoptosis [27, 28]. p-eIF2α suppresses global protein synthesis to conserve energy while facilitating gene reprogramming and the induction of key ER chaperones like BiP to restore protein homeostasis. IL-1β^low^ preconditioning further amplified the CYT-induced elevation of p-eIF2α levels in INS-1E cells (*p* < 0.05 vs. CYT; Fig. 5A, B) [14]. Both IL-1β^low^ preconditioning alone and followed by CYT stimulation led to a significant increase in BiP expression, as assessed by WB (*p* < 0.05 IL-1β^low^ vs. control; p < 0.01 IL-1β^low^ + CYT vs. CYT; Fig. 5C, D). *BiP* mRNA expression also increased following IL-1β^low^, requiring a 24 h washout period to return to baseline upon CYT stimulation *p* < 0.01 IL-1β^low^ + CYT vs. CYT; Fig. 5H).

In addition, IL-1β^low^ enhanced the expression of PDI, a chaperone essential for β-cell function (*p* < 0.01 vs. untreated), and restored the expression of chaperones downregulated by CYT, including GRP94 (*p* < 0.01), ORP150 (*p* < 0.05), and PDI (*p* < 0.01) (Fig. 5E–G). This IL-1β^low^-induced upregulation of key chaperones suggests an improved capacity for protein folding, processing, and secretion, potentially reinforcing β-cell resilience.

Regarding UPR sensor activation, CYT differentially affected the transcriptional activity of IRE1α-XBP1 and ATF6 pathways in INS-1E cells. While ATF6 transcriptional activity was reduced by CYT (*p* < 0.001), IRE1α-mediated XBP1 splicing remained unchanged, consistent with previous findings [14] (Fig. 5I, K). IL-1β^low^ treatment alone increased ATF6 transcriptional activity (*p* < 0.01 vs. control, Fig. 5J), but IL-1β^low^ preconditioning did not alter the transcriptional activity of ATF6 or IRE1α-mediated XBP1 splicing in response to CYT. XBP1s mRNA levels were downregulated by CYT; however, this reduction remained unaffected by IL-1β^low^ conditioning (Fig. 5I).

Overall, the INS-1E response to IL-1β^low^ suggests that, upon CYT stimulation, the induced proapoptotic ER stress is primarily regulated through the PERK/p-eIF2α pathway, leading to decreased expression of ATF4 (Fig. 4C), CHOP, and cleaved caspase-3 (Fig. 3H–J). Notably, IL-1β^low^ pre-treatment significantly attenuated CYT-induced expression of these proapoptotic drivers while simultaneously increasing the mRNA levels of antiapoptotic genes (Fig. 3E–G). IL-1β^low^-induced intracellular signaling highlights the PERK/p-eIF2α pathway as a key mediator of an adaptive response that helps preserve β-cell integrity.

### IL-1β^low^ attenuates CYT-induced downregulation of gene transcripts associated with β-cell identity/function, as well as the expression of Pdx-1 and insulin proteins

**Fig. 6:**
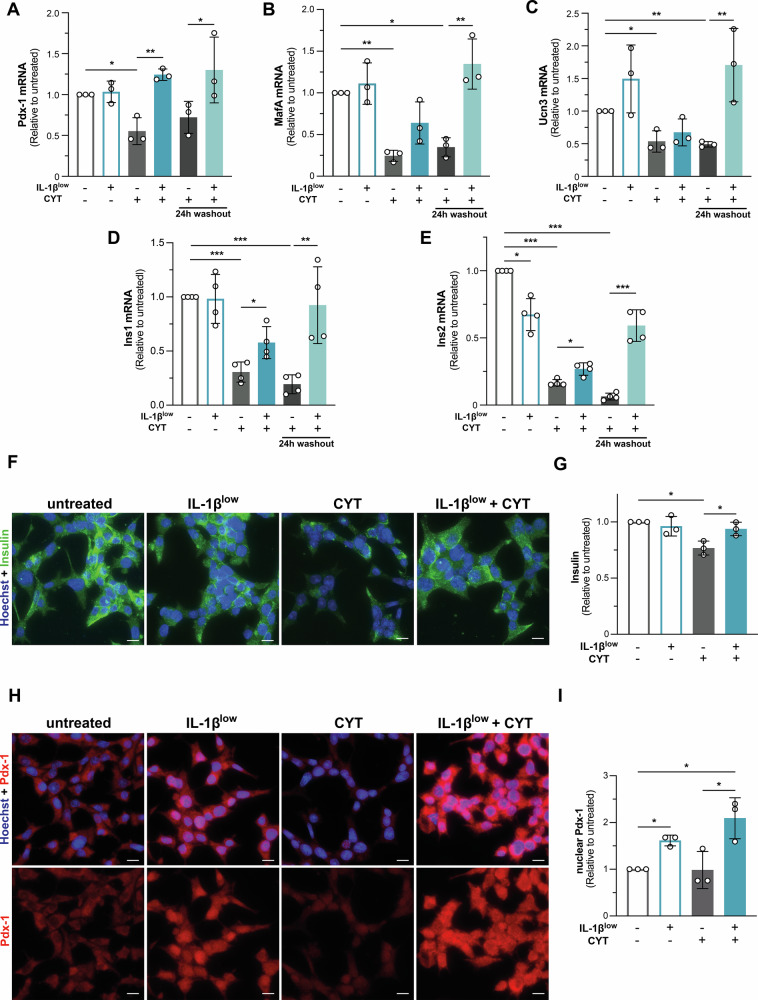
Effects of IL-1β^low^ on β-cell identity and function markers. **A**–**E** INS-1E cells were conditioned with IL-1β^low^ and subsequently challenged or not with CYT for 16 h. A 24 h washout period (without CYT) was also evaluated. **A** *Pdx-1* mRNA, **B** *Maf*A mRNA, **C** *Ucn3* mRNA, **D** *Ins1* mRNA, and **E** *Ins2* mRNA expression was analyzed by RT-qPCR. Relative mRNA levels were normalized to *HPRT*, *n* = 3–4. **F**–**I** INS-1E cells were preconditioned with IL-1β^low^ and subsequently challenged or not with CYT for 16 h. **F**, **G** Insulin (green) and **H**, **I** Pdx-1 (red) immunostaining were analyzed by fluorescence microscopy; nuclei were counterstained with Hoechst (blue). Scale bars = 10 μm. Quantifications were performed from the analysis of ten separate high-power fields per condition; *n* = 3. Data are shown as mean ± SD. (*) *p* < 0.05, (**) *p* < 0.01, (***) *p* < 0.001.

The harmful islet microenvironment during diabetes progression disrupts β-cell identity and maturity [29]. CYT stimulation reduced the expression of β-cell identity mRNA transcripts (*Pdx-1*, *MafA*, *Ucn3*, *Ins1/2*, *p* < 0.05 vs. untreated) in INS-1E cells (Fig. 6A–E). IL-1β^low^ mitigated these reductions, particularly for *Pdx-1*, *MafA* and *Ins1/2*, and enhanced their recovery, including *Ucn3* mRNA, after a 24 h washout.

In line with these findings, IL-1β^low^ prevented the CYT-induced reduction (23% vs. untreated) in immune-reactive insulin in INS-1E cells (*p* < 0.05 IL-1β^low^ + CYT vs. CYT; Fig. 6F, G). Nuclear localization of Pdx-1 immunoreactivity was enhanced by IL-1β^low^, both in the absence of challenge (*p* < 0.05 vs. untreated) and under CYT exposure (*p* < 0.05 vs. CYT; Fig. 6H, I).

**Fig. 7:**
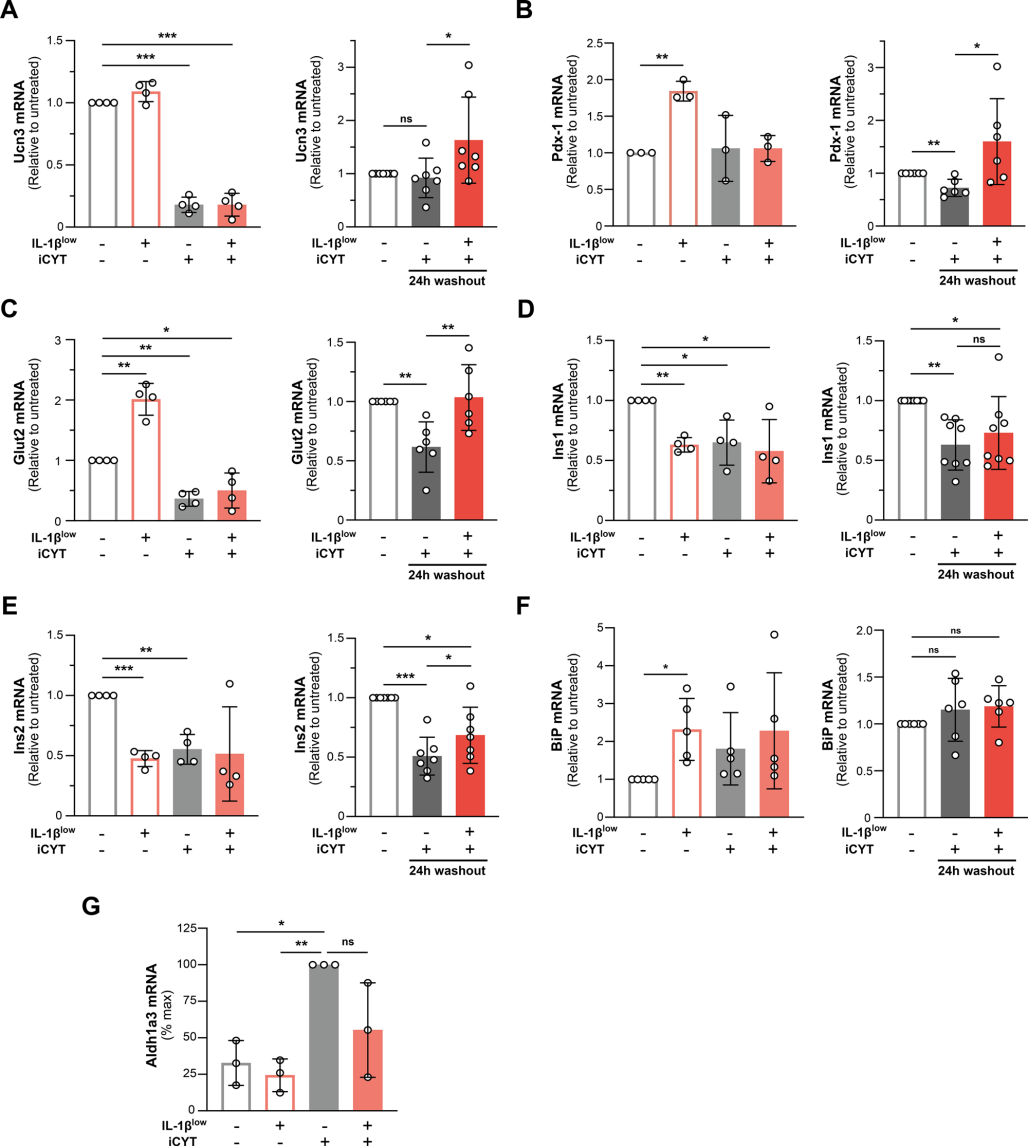
IL-1β^low^’s effects on gene transcripts associated with β-cell identity and function in murine islets. Murine islets (50 IEQ/well) were preconditioned with IL-1β^low^ and subsequently challenged or not with IL-1β 100 pg/ml + IFN-γ 5 ng/ml + TNF-α 8 ng/mL (iCYT) for 16 h. An additional condition involving a 24 h washout period (without iCYT) was also evaluated. **A** *Ucn3* mRNA, **B** *Pdx-1* mRNA, **C** *Glut2* mRNA, **D** *Ins1* mRNA, **E** *Ins2* mRNA, **F** *BIP* mRNA and **G** *Aldh1a3* mRNA expression were analyzed by RT-qPCR. Relative mRNA levels were normalized to *HPRT*, *n* = 3–8. Data are shown as mean ± SD (*) *p* < 0.05, (**) *p* < 0.01, (***) *p* < 0.001, (ns) not significant.

The pro-inflammatory cytokine mixture iCYT (IL-1β 100 pg/ml + IFN-γ 5 ng/ml + TNF-α 8 ng/ml) reduced mRNA expression of β-cell identity and functionality markers in isolated murine islets. IL-1β^low^ treatment facilitated their recovery in most cases, particularly after the washout period (Fig. 7A–F). IL-1β^low^ alone increased the expression of *Pdx-1*, *GLUT2* and *BiP* mRNA compared to untreated islets (Fig. 7B, C, F) and showed a trend toward mitigating the CYT-induced increase in the β-cell dedifferentiation marker *Aldh1a3* mRNA [30] (Fig. 7G). Collectively, the results indicate that IL-1β^low^ helps preserve β-cell identity in pancreatic islets under harmful CYT-induced stimuli.

### IL-1β^low^ enhances glucose-stimulated insulin secretion impaired by pro-inflammatory cytokines

To determine whether the beneficial effects of IL-1β^low^ on β-cells observed thus far translate into improved insulin secretion, we assessed glucose-stimulated insulin secretion (GSIS).

**Fig. 8:**
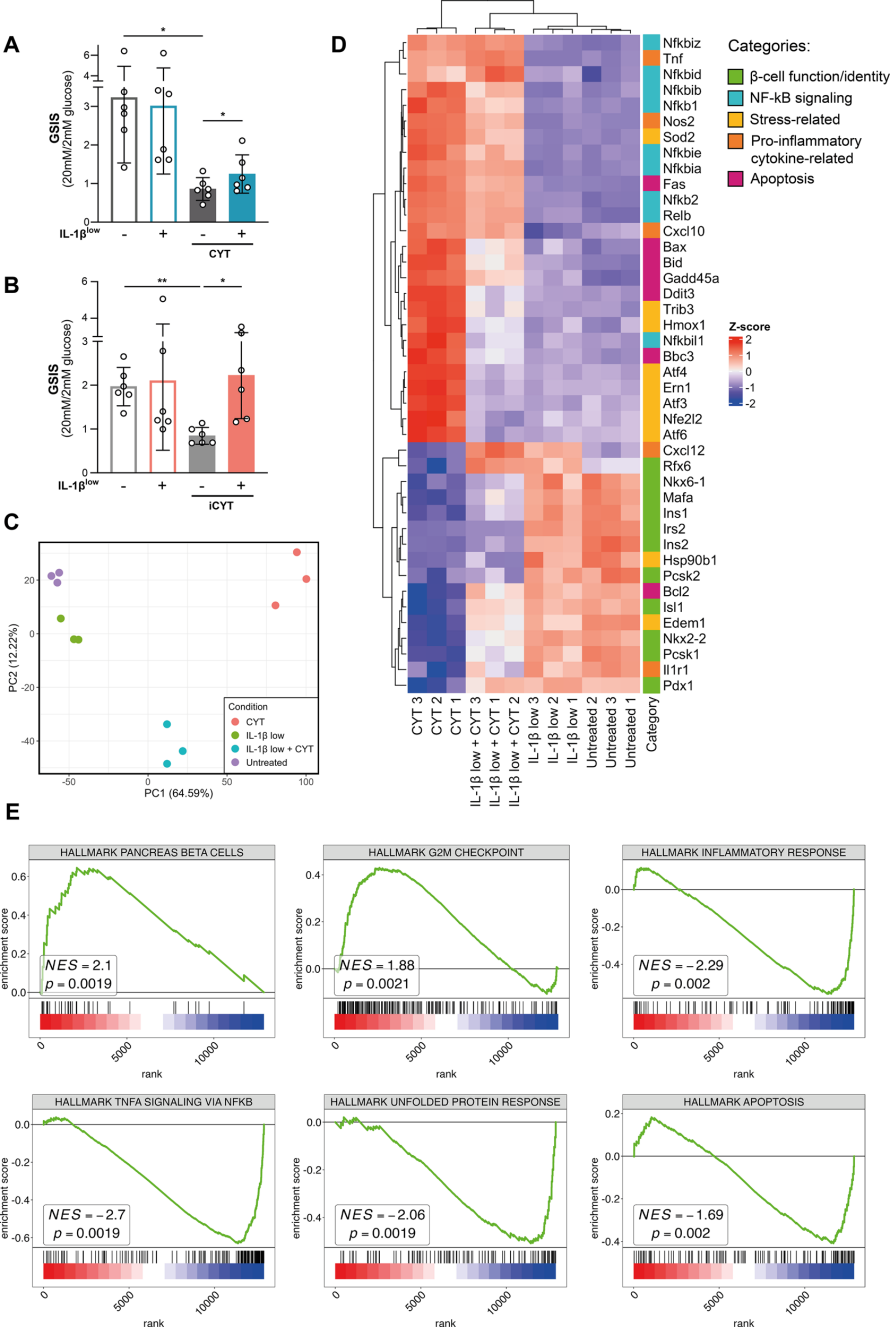
IL-1β^low^ helps preserve GSIS and modulates the transcriptomic profile. **A** INS-1E cells and **B** isolated mouse islets (5 IEQ/well) were preconditioned with IL-1β^low^ and then challenged or not with CYT (IL-1β 100 pg/ml + IFN-γ 5 ng/ml) or iCYT (IL-1β 100 pg/ml + IFN-γ 5 ng/ml + TNF-α 8 ng/ml), respectively. After 16 h, a glucose-stimulated insulin secretion (GSIS) assay was performed. Insulin levels in conditioned media were measured by ELISA. The insulin secretion index (20 mM/2 mM glucose) is expressed as mean ± SD, *n* = 6. (*) *p* < 0.05, (**) *p* < 0.01. **C**–**E** INS-1E cells were treated as in (**A**), and bulk RNA-seq was performed, *n* = 3. **C** Principal component analysis (PCA) of transcriptomic profiles shows distinct clustering by condition. Each point represents an individual sample, with the variance explained indicated on each axis. **D** Heatmap of log2CPM-normalized data (Z-score transformed), filtered to include differentially expressed genes (DEGs) identified from all pairwise comparisons. Colors indicate higher (red) and lower (blue) expression relative to the mean for a curated subset of these genes. Genes and samples were hierarchically clustered based on expression similarity. **E** Gene set enrichment analysis (GSEA) of IL-1β^low^-preconditioned INS-1E cells exposed to CYT, compared to CYT-challenged cells without preconditioning, using Hallmark gene sets: pancreatic β-cells, G2M checkpoint, inflammatory response, TNF-α signaling via NF-κB, unfolded protein response, and apoptosis. Normalized enrichment scores (NES) and p-values are shown for each pathway.

CYT exposure impaired GSIS (0.86 ± 0.29-fold vs. 3.2 ± 1.7-fold untreated, *p* < 0.05; Fig. 8A), an effect partially counteracted by IL-1β^low^ treatment in INS-1E cells (*p* < 0.05; Fig. 8A). IL-1β^low^ alone had no effect on insulin secretion. Remarkably, IL-1β^low^ restored GSIS in iCYT-challenged isolated islets, further supporting its role in improving islet health and enhancing insulin secretion (*p* < 0.05; Fig. 8B). Supplementary Fig. 3 presents the individual quantification of insulin secretion in response to low (2 mM) and high (20 mM) glucose for both INS-1 cells and isolated murine islets.

This finding is particularly significant as it underscores IL-1β^low^ ‘s protective role in preserving β-cell function in a cytokine-induced harmful environment, closely resembling the inflammatory microenvironment of islets in diabetes.

### RNA-seq reveals protective transcriptome modulation in IL-1β^low^-conditioned INS-1E cells challenged with cytotoxic cytokines

To identify the genetic mechanisms underlying phenotypic changes following IL-1β^low^ and CYT-treated cells conditioned with IL-1β^low^, we performed transcriptome analysis (Fig. 8C). We generated and sequenced bulk RNA-seq libraries from INS-1E cells subjected to these treatments, along with untreated and CYT-challenged cells as controls.

Principal component analysis reveals distinct clustering among experimental conditions, highlighting differences in transcriptional profiles. IL-1β^low^-treated cells exhibited a gene expression pattern similar to that of untreated cells. However, IL-1β^low^ preserved the expression of β-cell identity and functionality genes (*Rfx6*, *Nkx6-1*, *Ins1*, *Pdx-1*, *Mafa*, *Pcsk1*) in INS-1E cells that were subsequently exposed to a CYT challenge.

The CYT challenge induced significant transcriptional changes. However, IL-1β^low^-treated cells exhibited a distinct response to the proinflammatory challenge, displaying a transcriptional profile that set them apart from CYT-treated cells. IL-1β^low^ treatment downregulated apoptosis-related genes (*Fas*, *Ddit3*, *Bid*, *Bbc3*), attenuated the expression of genes associated with NF-κB signaling (*Nfkb2*, *Relb*, *Nfkb1*, *Nfkbil1*) and cellular stress (*Sod2*, *Trib3*, *Hmox1*, *Atf4*, *Ern1*, *Atf3*, *Atf6*, *Nfe2l2*), and reduced the expression of cytokine-related inflammatory genes (*Nos2*). The heatmap of selected genes further supports our qPCR findings (Figs. 1, 3, 6).

Gene Set Enrichment Analysis (GSEA) evaluated the enrichment of selected Hallmark Gene Sets in IL-1β^low^-treated cells challenged with CYT, compared to those receiving the CYT challenge (Fig. 8E). IL-1β^low^-treated cells showed positive enrichment in the pancreas beta-cell gene set, suggesting an upregulation of genes that support β-cell phenotype. Core enrichment genes, such as *Pcsk1* and *Isl1*, were identified as key contributors. A positive enrichment score for the G2M Checkpoint pathway suggests that IL-1β^low^-stimulated genes promote cell cycle progression, potentially enhancing proliferation or cell cycle regulation.

The UPR pathway exhibited negative enrichment in IL-1β^low^-treated cells, indicating reduced ER stress with *Atf3*, *Chac1*, and *Ern1* contributing to this effect. Other negatively enriched Hallmark gene sets included the inflammatory response, TNF-α signaling via NF-κB, and apoptosis pathways.

## Discussion

In this study, we show that IL-1β at basal physiological concentrations (IL-1β^low^), triggers a stress-response hormesis in vitro, strengthening pancreatic β-cell resilience and enhancing insulin secretion under inflammatory and cytotoxic conditions.

Priming β-cells with IL-1β^low^ activates survival mechanisms by modulating gene expression and promoting an adaptive response, reducing β-cell death/apoptosis triggered by pro-inflammatory cytokines in models mimicking the diabetic islet microenvironment. Interestingly, IL-1β^low^ did not induce a survival bias, as the number of cells remained unchanged after preconditioning, and no differences in cell death were observed between IL-1β-treated and control (untreated) cells.

β-cells exhibit a high density of IL-1R1 [31], prompting the question: why do these cells express abundant receptors that, when activated by their ligand, can initiate cell death? β-cells express components of the IL-1 signaling system, including IL-1α/β, IL-1R1, IL-1Ra, and IL-1R2, with the latter serving as a decoy receptor [25]. IL-1R1 knockout mice display impaired glucose tolerance and reduced insulin secretion [32]. Additionally, postprandial IL-1β secreted by macrophages contributes to blood glucose homeostasis [33]. Based on these observations, we investigated IL-1β priming’s effects on β-cells.

Prolonged inflammation disrupts the specialized phenotype of β-cells, leading to transdifferentiation and/or dedifferentiation [34]. This is clinically significant, as dedifferentiated β-cells are observed in patients with T1D and T2D, likely driven by chronic inflammation [35]. IL-1β (and/or TNF-α), combined with IFN-γ, disrupts cell function through NF-κB-regulated gene networks, ultimately leading to β-cell death. NF-κB-driven IL-1β transcriptional reprogramming reciprocally regulates chemokine and insulin secretion [36]. Cytokine-driven activation of the IKK complex triggers IκB phosphorylation, ubiquitination, and degradation, enabling NF-κB nuclear migration to induce inflammatory gene expression and mediators like iNOS [37, 38]. NO is a major driver of β-cell dysfunction and apoptosis [17, 18], impairs oxidative metabolism and insulin secretion, induces ER stress and activates signaling pathways culminating in β-cell apoptosis [11, 14]. However, depending on intracellular levels, NO can also suppress apoptosis via caspase-3-dependent DNA damage repair [20, 39–41].

We found that INS-1E cells preconditioned with IL-1β^low^ exhibited reduced NO secretion in response to CYT challenge (Fig. 1), mediated by suppression of the NF-κB signaling pathway, leading to decreased iNOS expression (Figs. 1, 2). Since CYT-induced NF-κB activation drives pro-apoptotic signaling in β-cells [14, 36], our findings suggest a protective role for IL-1β^low^ against CYT-induced cell death. We further assessed β-cell viability following IL-1β^low^ preconditioning and found that it significantly enhanced cell survival after both short-term and long-term CYT exposure (Fig. 3). A previous study reported a cytoprotective effect of IL-1β on β-cells. Research involving rat β-cells indicated that IL-1β may protect against necrosis caused by STZ or alloxan, although it did not protect against cytokine-induced apoptosis. This protection, however, came at the cost of β-cell phenotype integrity, mediated through an NO-dependent mechanism [42]. This apparent discrepancy with our results may be explained by the differences in experimental conditions. In this study, β-cells were exposed to very low concentrations (∼10 pg/ml) of IL-1β for 72 h. In contrast, the referenced study used higher IL-1β concentrations over a shorter exposure period.

While some studies dismiss a direct link between CHOP and cytokine-induced β-cell death [43], others highlight its key role in CYT-induced apoptosis, as its knockdown significantly reduces this effect in INS-1E cells [37]. Additionally, studies suggest that CHOP also has a pro-inflammatory function [44]. Consistently, CYT challenge led to increased CHOP expression levels. However, in IL-1β^low^-conditioned INS-1E cells, both CHOP and cleaved caspase-3 were downregulated. These findings align with previous research identifying CHOP as a key regulator of β-cell apoptosis. CHOP contributes to CYT-induced NF-κB-dependent pathways (*e.g*., NO production, induction of pro-apoptotic mediators) and regulates mitochondrial-mediated apoptosis (e.g., caspase-3) [37]. In addition to confirming CHOP’s relevance as a mediator in CYT-induced activation of the intrinsic apoptotic pathway, we provide new insights into how IL-1β^low^ attenuation of CHOP impacts β-cell survival under CYT/inflammatory challenge.

The UPR preserves cellular homeostasis under stress; however, excessive or prolonged ER stress compromises β-cell function and survival. β-cells rely on the ER and UPR machinery to process excess nutrients and ensure proper insulin folding and secretion [45]. Unresolved UPR contributes to T1D and T2D [3, 46]. The UPR cascade is initiated upon BiP dissociation by the autophosphorylation of PERK and IRE1, along with the proteolysis of ATF6 [47]. While XBP1 and ATF6 manage ER stress, their reduced expression may limit adverse effects, support metabolic adaptation, and mitigate inflammation. However, persistent ER stress can still lead to ATF4-mediated CHOP activation [48].

Cytokine-induced ER stress shifts β-cell energy priorities, promoting survival mechanisms at the expense of normal cellular functions, including protein folding, synthesis, and insulin secretion. However, exacerbated ER stress activates PERK/eIF2α/ATF4/CHOP pathway leading to β-cell dysfunction and apoptosis [1]. The PERK/p-eIF2α pathway plays a crucial role in cell survival under stress by reducing global protein synthesis while selectively translating specific mRNAs, such as ATF4, which can drive either pro-survival or pro-apoptotic responses depending on the cellular context [49].

Notably, IL-1β^low^ enhanced CYT-induced eIF2α phosphorylation in INS-1E cells, accompanied by reduced ATF4 and CHOP protein levels (Fig. 5). At the same time, IL-1β^low^ favored the expression of ER chaperones crucial for insulin folding, processing, and handling, such as BIP [50] and GRP94 [51], respectively. These findings suggest that IL-1β^low^ induces a distinct adaptive response and that its precise molecular mechanism requires further investigation. However, it clearly contributes to preserving β-cell integrity under pro-inflammatory cytokine-induced ER stress, with the pro-survival effects of the PERK/p-eIF2α pathway mediated through mechanisms involving ATF4/CHOP downregulation, although additional protective mechanisms cannot be ruled out. Future experiments should determine whether IL-1β^low^-induced PERK/p-eIF2α signaling promotes survival by reducing oxidative stress or enhancing autophagy while avoiding ATF4/CHOP upregulation [49, 52].

IL-1β^low^ mitigated the CYT-induced decline in β-cell identity and maturity markers (*e.g*., *Ucn3, MafA, Pdx-1*, and *GLUT2*) in both INS-1E cells and mouse islets (Figs. 6, 7) while preserving Pdx-1 and insulin expression (Fig. 6). This contrasts with previous reports suggesting that low concentrations of IL-1β drive β-cell dedifferentiation and dysfunction [53], possibly due to variations in its concentration and exposure duration.

IL-1β^low^ improved β-cell insulin secretion despite the acute impairment of glucose-stimulated insulin release by pro-inflammatory cytokines [9, 11, 14, 42, 54], with a stronger effect in isolated murine islets. This suggests IL-1β^low^ may also support other islet-resident cells, warranting further investigation into its broader islet benefits.

RNA-seq revealed a protective transcriptomic profile in IL-1β^low^-preconditioned INS-1E cells under CYT stimulation. DEGs showed preserved β-cell identity and reduced expression of inflammation, NF-κB signaling, ER stress, and apoptosis-related genes. IL-1β^low^ and IL-1β^low^ + CYT cells exhibited increased *Rfx6* gene transcript expression, encoding a protein essential for islet cell development and insulin production [55]. Mutations in *Rfx6* are associated with maturity-onset diabetes of the young [56], and its expression is dysregulated in human β- and α-cells in both T1D and T2D [57], while β-cell-specific *Rfx6* knockout mice exhibit impaired insulin secretion [58]. Isl1 regulates genes essential for β-cell differentiation and maturation, such as *Pdx-1* and *Slc2a2* (*GLUT2*) vital for β-cell function and glucose sensing, respectively [59]. IL-1β^low^-treatment preserved *Isl1* expression in INS-1E cells. The ER chaperone Edem1 supports insulin processing and β-cell function by mitigating ER stress [60]. Additionally, GSEA indicates that IL-1β^low^ may enhance β-cell resilience by upregulating the cell cycle under inflammatory conditions. The enrichment of the G2M Checkpoint pathway may reflect an adaptive mechanism that counterbalances stress-induced β-cell loss by enhancing proliferative capacity or reinforcing cell cycle control. The observed negative enrichment in the UPR, inflammatory response, and apoptosis pathways indicates that IL-1β^low^ may reduce ER stress and inflammatory signaling, contributing to improved β-cell survival. The downregulation of genes involved in inflammation and cell death aligns with a protective role for IL-1β^low^ in modulating stress responses, ultimately fostering β-cell adaptation in a pro-inflammatory environment. qPCR validation of DEGs and GSEA-enriched genes in IL-1β^low^-treated cells is needed given their role in β-cell identity, function, and survival.

Collectively, we describe a novel aspect of IL-1β^low^’s effects on β-cells, highlighting its ability to induce gene expression changes, modulate ER stress and UPR. These changes enhance cellular resilience against inflammatory cytotoxic challenges triggered by cytokines. However, several questions remain to be addressed: 1) Does β-cell resilience result from a single adaptive molecular pathway in response to IL-1β^low^? 2) Could β-cell resilience be induced by other stress-inducing agents? 3) Since IL-1β^low^ induces an increase in p-eIF2α levels, and mammalian stress granules (SGs) are known to assemble in response to stress-induced p-eIF2α [61], could the β-cell response to IL-1β^low^ be associated with the protective effect mediated by SGs formation? and 4) Could IL-1β^low^’s effects be replicated in vivo?

Given that individuals genetically predisposed to T1D, T2D, obesity, or metabolic syndrome do not always progress to overt diabetes, it is plausible that, under certain conditions, β-cells activate protective defense mechanisms [6]. Our findings suggest that mild or transient stress induced by IL-1β^low^ can trigger such protective responses. Future research should focus on identifying novel hormesis inducers (hormetins) in β-cells and uncovering their mechanisms to develop therapies that enhance β-cell function and survival in diabetes.

## Materials and methods

### Reagents

Culture media, supplements and antibiotics were purchased from Gibco (Thermo Fisher Scien- tific, Carlsbad, CA, USA). Fetal bovine serum was obtained from Natocor (Córdoba, Argentina). Recombinant cytokines were purchased from R&D Systems (Minneapolis, MN, USA). Tauroursodeoxycholic acid (TUDCA), 5-methylisothiourea sulfate (SMT) and other analytical-grade reagents were purchased from Sigma-Aldrich.

### Animals

C57BL/6NCrl mice were bred in a controlled environment (20–22 °C, 12 h light–dark cycle) at the IIMT (Austral University-CONICET) animal facility and given *ad libitum* access to food and water. All procedures were conducted in accordance with the Guide for the Care and Use of Laboratory Animals, Eighth edition (2011). The study was approved by the Animal Research and Care Committee (CICUAL #2023-03) at Austral University.

### INS-1E cell line

The rat β-cell line INS-1E (Prof. Wollheim, University Medical Centre, Geneva, Switzerland) was used between passages 63 and 90, and cultured at 37 °C in a humidified atmosphere containing 5% (vol./vol.) CO2 in complete RPMI 1640 culture medium [11 mM glucose, 10% (vol./vol.) heat-inactivated fetal bovine serum (FBS), penicillin (50 IU/ml), streptomycin (50 μg/ml), L-glutamine (2 mmol/l), 2-mercaptoethanol (50 μmol/l), HEPES (10 mmol/l) and sodium pyruvate (1 mmol/l)]. The presence of mycoplasma was periodically checked by PCR. INS-1E were seeded at a density of 11 × 10^3^ cells/cm^2^ in multiwell plates (Nunc, Thermo Scientific, Denmark) in complete medium.

### Mice islets isolation and culture

Islets (C57BL/6NCrl) were isolated by collagenase digestion and handpicked after density gradient centrifugation [62]. For standardization purposes, islets with a diameter of 100–125 μm were defined as one islet equivalent (IEQ). Islets were cultured on ultra-low attachment plates (Corning Costar, Kennebunk, ME, USA), at 37 °C in humidified atmosphere containing 5% (vol./vol.) CO2 in RPMI 1640 medium containing 5.5 mM glucose, 10% FBS, penicillin (50 IU/ml), streptomycin (50 μg/ml), L-glutamine (2 mmol/l) and HEPES (10 mmol/l) for 24 h prior to performing experiments.

### Hormesis induction by IL-1β treatment

INS-1E cells were conditioned with IL-1β 10 pg/ml for 72 h (IL-1β^low^), with fresh cytokine added every 24 h without replacing the culture media. Then, the culture media was renewed, and cells were challenged with a proinflammatory cytokine mixture (CYT: IL-1β 100 pg/ml + IFN-γ 5 ng/ml). When INS-1E cells were allowed to recover, CYT-containing media was removed after 16 h, followed by PBS washing and a 24 h incubation in CYT-free RPMI with 10% FBS before harvesting (24 h washout).

Mouse islets were treated with IL-1β, similar to INS-1E cells. After 72 h, the culture medium was refreshed, and the islets were challenged with a proinflammatory cytokine mixture (iCYT: IL-1β 100 pg/ml, IFN-γ 5 ng/ml, TNF-α 8 ng/ml) for 16 h. For recovery, the iCYT-containing medium was removed, and the islets cultured for 24 h, as with INS-1E cells. Alternatively, for GSIS experiments, islets were treated with IL-1β 10 pg/ml every 72 h, with media replaced each time IL-1β was added. After three IL-1β treatments, islets were challenged with iCYT for 16 h before starting the GSIS protocol.

### SDS-PAGE and Western blot

INS-1E cells were harvested on ice-cold PBS, washed and lysed in lysis buffer [50 mM Tris–HCl pH 7.4, 250 mM NaCl, 25 mM NaF, 2 mM EDTA, 0.1% Triton-X, protease inhibitors mix (Complete ULTRA, Roche)]. Protein concentration was determined using the BCA assay Kit (Pierce, Thermo Fisher Scientific, Carlsbad, CA, USA) and samples were stored at −20 °C. Proteins were separated by 8–12% SDS-polyacrylamide gel electrophoresis (SDS-PAGE), blotted onto nitrocellulose or PVDF membranes (GE-Healthcare, Amersham, UK) and incubated with primary antibodies: IκBα (#4814, 1:1000), p-IκBα (#9246, 1:1000), β-actin (#3700, 1:1000), ATF4 (#11815, 1:1000), CHOP (#2895, 1:1000), Cleaved caspase-3 (#9664, 1:1000), eIF2α (#2103, 1:1000), p-eIF2α (#9721, 1:1000), PDI (#3501, Cell Signaling Technology, Danvers, MA, USA, 1:1000); iNOS (#610332, BD Biosciences, San Jose, CA, USA, 1:1000), ORP150 (#ab124884, 1:1000), GRP94 (#ab13509, 1:1000), BIP (#ab21685, Abcam, Cambridge, MA, USA, 1:1000). Blots were incubated with HRP-conjugated secondary antibodies: Goat anti-Mouse IgG (H + L) (#62-6520; Thermo Fisher Scientific, Carlsbad, CA, USA, 1:5000) and Goat Anti-Rabbit IgG (H + L) (#BA1054, Boster Biological Technology, Pleasanton, CA, USA, 1:5000), followed by visualization using ECL (Supersignal; Thermo Fisher Scientific, Carlsbad, CA, USA).

### Immunofluorescent microscopy

INS-1E were cultured for 72 h on fibronectin-coated coverslips, treated as described in the figures, fixed by cold methanol and incubated with primary antibodies: monoclonal mouse anti-insulin (clone HB125); NFκB p65 (RelA, #sc-109, Santa Cruz Biotechnology, 1:60) or Pdx-1 (#5679, Cell Signaling Technology, Danvers, MA, USA, 1:100). Secondary antibodies were used at a 1:200 dilution: anti-mouse Alexa Fluor 488 or anti-rabbit Alexa Fluor 647 conjugated dye (Thermo Fisher Scientific, Carlsbad, CA, USA). Coverslips were mounted on slides with Mowiol and images were acquired on a NIKON Eclipse Ni microscope (Nikon, Tokyo, Japan). Image quantification was performed with Fiji software.

### Nitric oxide quantification

Nitrite levels were measured as an indicator of nitric oxide (NO) production using the Griess reagent (1% sulfanilamide and 0.1% naphthyl ethylenediamine dihydrochloride in 2.5% phosphoric acid) at 570 nm [14].

### Quantitative real-time PCR

Total RNA was extracted from INS-1E cells using TRIzol reagent (Thermo Fisher Scientific, Carlsbad, CA, USA) following the manufacturer’s instructions. Nucleic acid quantification and quality control were assessed with a NanoDrop One spectrophotometer (Thermo Fisher Scientific, Carlsbad, CA, USA). For cDNA synthesis, 1 μg of RNA was reverse-transcribed using RevertAid Reverse Transcriptase in the presence of RiboLock RNase Inhibitor (Thermo Fisher Scientific, Carlsbad, CA, USA) and oligo(dT) primers. All primers were designed using Primer3 and BLAST (NIH) (Supplementary Table 1). Real-time PCR was performed on an AriaMx Real-Time PCR Detection System (Agilent Technologies, Santa Clara, CA, USA), using Master Mix qPCR 2.0 Sybr Rox (Embiotec, BA, Argentina). Each reaction was carried out in triplicate, using HPRT as the normalization control. Relative gene expression was determined by the 2 − ΔΔCT method.

### Transient transfections and luciferase reporter assays

NF-κB transcriptional activity was evaluated by transfecting INS-1E cells with a plasmid containing multimerized NF-κB-binding sites linked to a minimal promoter upstream of the luciferase gene (κB-Luc promoter) [63]. ATF6 pathway activation was assessed using a reporter plasmid in which the firefly luciferase gene is driven by five copies of the ATF6 consensus binding site (5xATF6-LUC). To quantitatively measure XBP1 splicing, we employed a splicing-specific reporter plasmid where the firefly luciferase coding sequence is fused to the second ORF of unspliced XBP1 (XBP1u-LUC); luciferase expression occurs only upon IRE1-mediated splicing that removes the 26-nt intron. All transfections included a CMV-Renilla LUC expression vector for normalization.

Plasmids were transfected into INS-1E cells using Lipofectamine 3000 reagent (Thermo Fisher Scientific) in Opti-MEM medium following IL-1β^low^ conditioning. Thirty hours post-transfection, cells were challenged with pro-inflammatory cytokines. After treatment, cells were lysed and firefly and Renilla luciferase activities were sequentially measured using the Dual-Glo Luciferase Reporter Assay System (Promega) on a Centro LB963 luminometer (Berthold, Germany).

### Assessment of cell viability and apoptosis

For cell viability assays, INS-1E cells were seeded in 96-well plates. After treatment, the medium was replaced with fresh medium containing 0.5 mg/mL MTT (Thermo Fisher Scientific, Carlsbad, CA, USA). After 3 h at 37 °C, the medium was removed and replaced with 100 μL of acidified isopropanol (40 mM HCl), followed by incubation at room temperature for 15 min. Absorbance was measured at 570 nm [14].

For apoptosis assessment, INS-1E cells were seeded onto fibronectin-coated coverslips and treated as described in the figures. After treatment, cells were washed and stained with Hoechst 33342 (20 μg/ml) and propidium iodide (PI; 20 μg/mL) for 30 min at 37 °C. Coverslips were mounted on slides with Mowiol, and images were immediately acquired using a NIKON Eclipse Ni microscope (Nikon, Tokyo, Japan). The percentage of apoptotic cells was analyzed by two investigators blinded to the experiment using Fiji software. Additionally, apoptosis was evaluated by phosphatidylserine exposure analysis using PE-Annexin V and 7-AAD staining (BD Biosciences) according to the manufacturer’s instructions, followed by flow cytometry analysis (BD Accuri C6 Plus).

## Insulin quantification and glucose-stimulated insulin secretion (GSIS)

Insulin secretion from INS-1E and islets was quantified using a sandwich ELISA [14]. For GSIS, cells/islets were incubated in Krebs–Ringer phosphate buffer (KRB: 135 mmol/l NaCl, 0.5 mmol/L NaH2PO4, 3.6 mmol/l KCl, 0.5 mmol/L MgCl2, 1.5 mmol/L CaCl2, 5 mM NaHCO3, pH 7.4) supplemented with 10 mmol/L HEPES and 0.1% BSA. Cells/islets were first incubated in glucose-free medium for 2 h, followed by a 1-h incubation in fresh KRB-HEPES-BSA containing 2 mmol/l glucose. The supernatant was discarded, and cells/islets were incubated again in fresh KRB-HEPES-BSA with 2 mmol/L glucose. The supernatant was collected, and the cells/islets were subsequently incubated in KRB-HEPES-BSA with 20 mmol/l glucose for an additional 1 h before collecting the solution. Secreted insulin was normalized to total protein content in cell/islet lysates and stimulation index was calculated as the ratio of insulin released under high glucose versus low glucose condition. Protein concentration was determined using the BCA assay Kit (Pierce).

### RNAseq and bioinformatic analysis

Total RNA was extracted from INS-1E cells, and RNA sequencing (RNA-seq) libraries were prepared using the TruSeq RNA Library Prep Kit (Illumina). Sequencing was performed on the Illumina platform. Analyses were conducted in RStudio (R version 4.3.3) using Bioconductor packages. Raw sequencing reads underwent quality control using FastQC (version v0.11.9) to assess read quality [64]. Preprocessing, including adapter trimming and filtering of low-quality reads was performed using the rfastp package (version 1.12.0) [65]. The rat reference genome (mRatBN7.2, assembly GCF_015227675.2) was downloaded from NCBI and used to build a reference index with the Rsubread package (version 2.16.1) [66]. This index was subsequently used for read alignment. Read quantification was carried out with the featureCounts function from the Rsubread package, using the Rattus norvegicus gene annotation file (mRatBN7.2 GTF). The resulting count matrix was exported for further statistical analysis.

The edgeR package (version 4.0.16) was used to normalize sequencing counts and perform differential expression analysis between selected conditions [67]. To filter out lowly expressed genes, only genes with counts per million (CPM) > 1 in at least two samples were retained. Library sizes were recalculated, and normalization was performed using TMM (trimmed mean of M-values) normalization. Dispersion estimation was conducted, followed by the generation of a biological coefficient of variation (BCV) plot to assess variability across samples. For variance stabilization, the normalized expression data derived from the RNA-seq count matrix was voom-transformed using the limma package (version 3.58.1) [68]. The transformed matrix was subsequently used for principal component analysis (PCA).

The normalized count matrix was log2-transformed (log2-CPM) for heatmap generation. The heatmap was generated from a pre-filtered count matrix based on a list of differentially expressed genes (DEGs) that included all pairwise comparisons performed. DEGs were identified using exactTest, with different thresholds depending on the comparison: in IL-1β^low^-treated cells versus untreated cells, differentially expressed genes were selected using a false discovery rate (FDR) < 0.01 and |log2 fold change (log2FC)| > 0.6. This more permissive threshold was used because the untreated and IL-1β^low^-treated samples were highly similar, and the small differences between them required a less stringent log2FC cutoff to allow for the selection of statistically significant genes with low variation in expression. For all other comparisons, a threshold of FDR < 0.01 and |log2FC| > 1 was applied.

GSEA (Gene Set Enrichment Analysis) was conducted using the msigdbr package (version 10.0.1) to obtain gene sets from the Hallmark Gene Set specific to the rat species (*Rattus norvegicus*). The ranked gene list, based on the log fold change (logFC) was used to perform the enrichment analysis with the fgsea package (version 1.28.0). The analysis aimed to identify pathways with significant positive or negative enrichment. Results were filtered to retain only those meeting a statistical significance threshold of *p* < 0.05. The gggsea package was used to visualize the GSEA results.

### Statistical analysis

Results are presented as mean ± SD. Comparison between groups was carried out using paired or unpaired Student ´s *t* test or ANOVA followed by Bonferroni ´s multiple comparison test, as appropriate. A *p* < 0.05 was considered to indicate a statistically significant difference. All statistical analyses were performed using GraphPad Prism version 10.2.3 Software.

## Supplementary information

supplemental material

uncropped original western blots

## Data Availability

RNA-seq data have been deposited in NCBI’s Gene Expression Omnibus and are accessible through GEO Series accession number GSE305828. The original data of Western blots are all shown in Supplementary Original Data. All other data generated or analyzed during this study are available from the corresponding author upon reasonable request.
