# Supplementary material for: IL-1β priming triggers an adaptive stress response that enhances pancreatic β-cell resilience to subsequent cytotoxic inflammatory insult
Source: Cell Death Dis. 2025 Oct 21;16(1):744. doi: 10.1038/s41419-025-08059-0 (PMC12540857; doi:10.1038/s41419-025-08059-0)
Supplement: Supplementary file 1 — supplemental material [file 41419_2025_8059_MOESM1_ESM.pdf]

Supplementary Information (SI)

Cell Death and Disease

IL-1β priming triggers an adaptive stress response that enhances pancreatic β-cell resilience to subsequent cytotoxic inflammatory insult

Carolina Sétula[1,2], Ingrid Pensado-Evans[1,2], Andrea Scelza-Figueredo[1,2]*, Miranda Sol Orellano[1,2]*, Ignacio Rodríguez-Valero[1,2], Eduardo Spinedi[3], Raghavendra G. Mirmira[4,], Luz Andreone[1,2] and Marcelo Javier Perone[1,2‡]

[1] Immuno-Endocrinology, Diabetes & Metabolism Laboratory, Instituto de Investigaciones en Medicina Traslacional (IIMT), CONICET-Universidad Austral, Pilar, Argentina.
[2] Facultad de Ciencias Biomédicas, Universidad Austral, Pilar, Argentina.
[3] Centre of Experimental and Applied Endocrinology (CENEXA, UNLP-CONICET), La Plata Medical School, Universidad Nacional de La Plata, Argentina.
[4] Department of Medicine and the Kovler Diabetes Center, The University of Chicago, Chicago, Illinois, USA.

Supplementary Table 1. Primer sequences for qPCR

Gene name	Forward primer	Reverse primer	Target species
iNOS	CAGCTGGGCTGTACAAACCTT	CATTGGAAGTGAAGCGTTTCG	rat
IL-1β	ACAAAAATGCCTCGTGCTGTC	GTGCCGTCTTTCATCACACAG	rat
IL-1β	TTGAAGTTGACGGACCCCAA	ATGTGCTGCTGCGAGATTTG	mouse
IL-1Ra	CATCCTTCTGTTTCGTTCAGAGTC	GAGCTGGTTGTTCCTCAGGTAG	rat
IL-1Ra	GGAAAAGACCCTGCAAGATGC	GGATGCCCAAGAACACACTAT	mouse
IL-1R1	ACTCCTGCTCTGATTTTCTTCC	TCCAAACTGTCCCTCCAAGACC	rat
IL-1R2	GTGTGACTGAGGGGCTACAC	CGTGGATTCGTGGCAACAC	rat
DP5	GCCGTGGTGTTACTTGGA	GATTGTGCCAGAGCTTCACA	rat
PUMA	AGTGCGCCTTCACTTTGG	CAGGAGGCTAGTGGTCAGGT	rat
Bax	GGCTGGACACTGGACTTCCT	GGTGAGGACTCCAGCCACAA	rat
Bcl-2	GATGACTGAGTACCTGAACCG	CAGAGACAGCCAGGAGAAATC	rat
XBP1s	GAGTCCGCAGCAGGTG	GCGTCAGAATCCATGGGA	rat
XBP1t	CACAGACTGCGCGAGATAGA	CATCCCCAAGCGTGTCCTTA	rat
Pdx-1	CCCGAATGGAACCGAGACTG	TGTAGGCTGTACGGGTCCTC	rat, mouse
MafA	AAGCCGAGGAACTTACCTGC	TAGAGAAATAGCAGCGCGGG	rat
Ucn3	GGAGTGGAGCGGTTTCCATA	CATCAGCATCGCTCCCTGTA	rat, mouse
Ins1	ACCTTTGTGGTCCTCACCTG	AGCTCCAGTTGTGGCACTTG	rat
Ins2	TGTGGTTCTCACTTGGTGGA	CTCCAGTTGTGCCACTTGTG	rat
Ins1	TGGACTATAAAGCTGGTGGGC	TTGAAACAATGACCTGCTTGC	mouse
Ins2	AGGACCCACAAGTGGCACAA	GGTAGGCTGGGTAGTGGTGG	mouse
Glut2	CTGGAGCCCTCTTGATGGG	CCAGTCCTGAAATTAGCCCAC	rat, mouse
Aldh1a3	CCCTTCGATGCCAAAACGG	CATTCTAGCTTGGCCCCTTC	mouse
BiP	ACCACCTATTCCTGCGTCG	GGTTGGACGTGAGTTGGTTC	rat, mouse
HPRT	AGTCCCAGCGTCGTGATTAGCG	GGGCCACAATGTGATGGCCTCC	rat, mouse

Supplementary figure 1

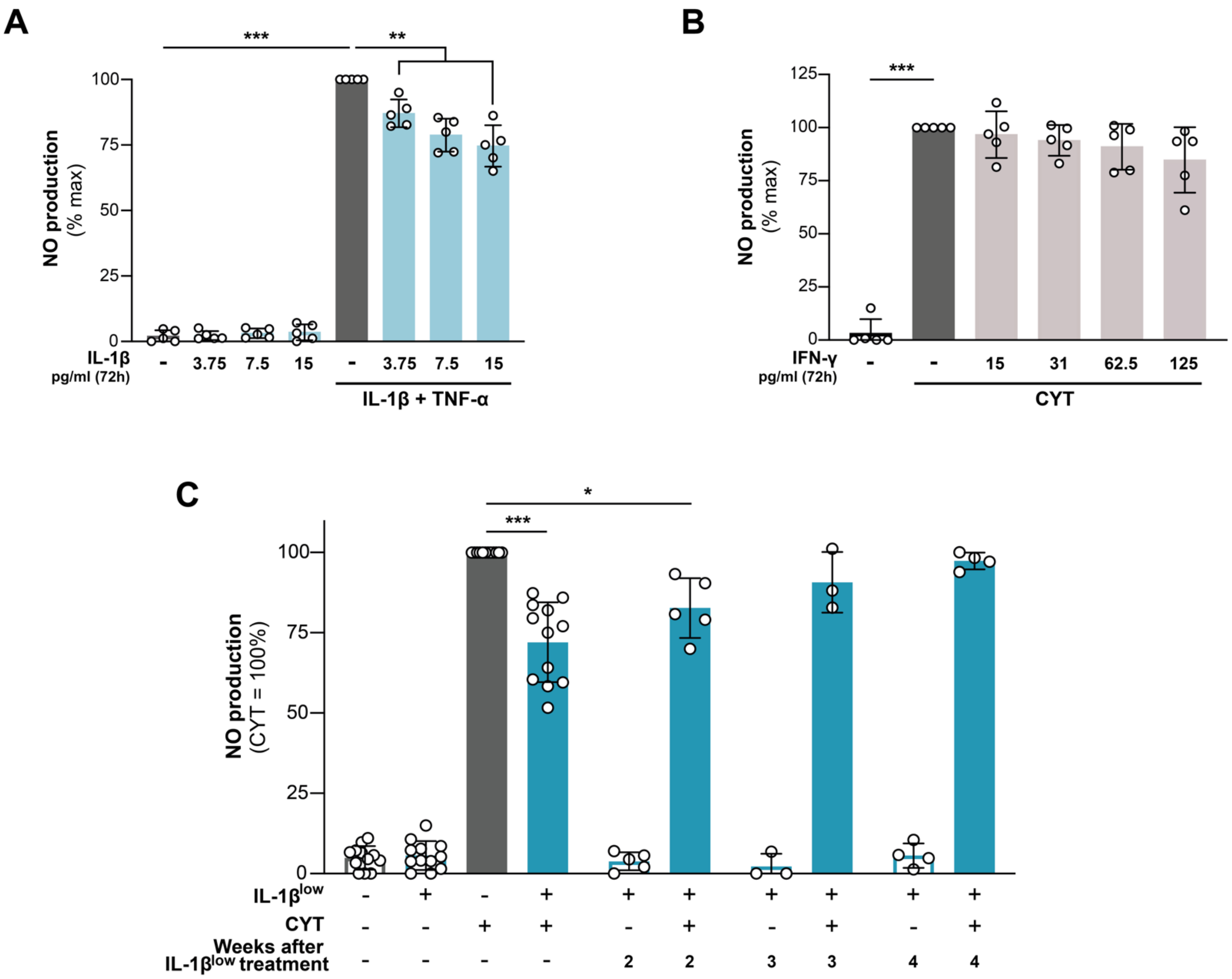

Supplementary Figure 1: A. INS-1E cells were treated for 72 h with IL-1β (3.75, 7.5 or 15 pg/ml as indicated) and subsequently challenged or not with IL-1β 100 pg/ml + TNF-α 8 ng/ml for 16 h, n=5.

B. INS-1E cells were treated for 72 h with IFN-γ (15, 31, 62.5 or 125 pg/ml as indicated) and subsequently challenged or not with or IL-1β 100 pg/ml + IFN-γ 5 ng/ml (CYT) for 16 h, n=5.

C. INS-1E cells were conditioned with IL-1β^{low} and subsequently challenged or not with CYT for 16h. The CYT challenge was applied either immediately after IL-1β^{low} treatment, or after 2, 3 or 4 weeks, n=3-5. During these intervals, cells were trypsinized two, three or four times, respectively. NO levels in the conditioned media were assessed by Griess reaction and normalized to total cell protein content. Data are shown as mean ± SD. (*) $p < 0.05$, (**) $p < 0.01$, (***) $p < 0.001$.

Supplementary figure 2

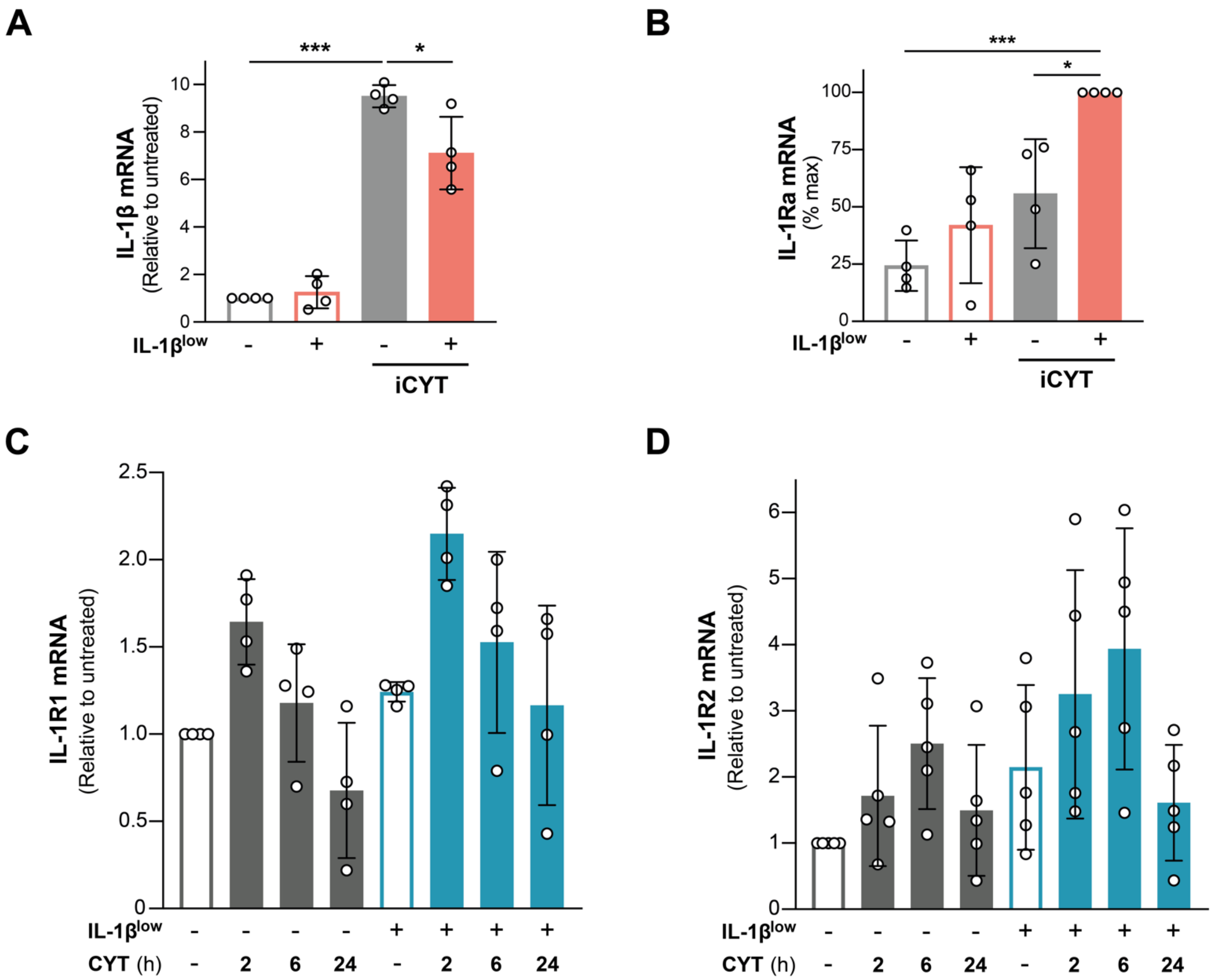

Supplementary Figure 2: A-B. Murine islets (50 IEQ/well) were conditioned for 72 h with IL-1β 10 pg/ml (IL-1β^{low}) and subsequently challenged or not with IL-1β 100 pg/ml + IFN-γ 5 ng/ml + TNF-α 8 ng/mL (iCYT) for 16h. **A.** *IL-1β* mRNA and **B.** *IL-1Ra* mRNA expression were analyzed by RT-qPCR. Relative mRNA levels normalized to *HPRT*, n=4. **C-D.** INS-1E cells were conditioned for 72 h with IL-1β 10 pg/ml (IL-1β^{low}) and subsequently challenged or not with IL-1β 100 pg/ml + IFN-γ 5 ng/ml (CYT) for the indicated times. **C.** *IL-1R1* mRNA and **D.** *IL-1R2* mRNA expression were analyzed by RT-qPCR. Relative mRNA levels normalized to *HPRT*, n=4-5. Data are shown as mean ± SD. (*) $p < 0.05$, (***) $p < 0.001$.

Supplementary figure 3

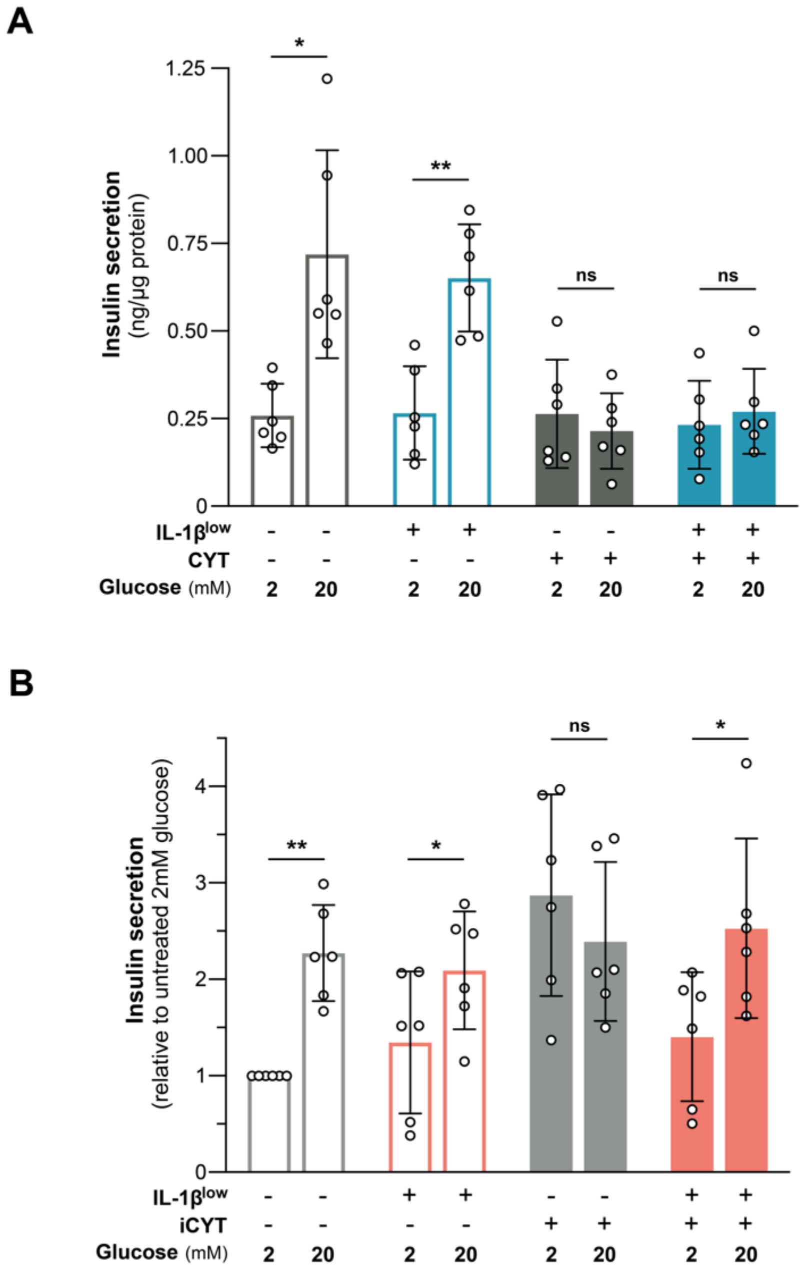

Supplementary Figure 3: A. INS-1E cells and **B.** isolated mouse islets (5 IEQ/well) were preconditioned with IL-1β^{low} and then challenged or not with CYT (IL-1β 100 pg/ml + IFN-γ 5 ng/ml) or iCYT (IL-1β 100 pg/ml + IFN-γ 5 ng/ml + TNF-α 8 ng/ml), respectively. After 16h, a glucose-stimulated insulin secretion (GSIS) assay was performed. Insulin levels in the conditioned media of cells or islets cultured in low (2mM) or high (20 mM) glucose were measured by ELISA and normalized by total protein content. **A.** Insulin levels are presented as ng/µg protein, n=6. **B.** Due to inter-experimental variability in absolute insulin levels, insulin secretion is shown relative to that of untreated islets under 2mM glucose stimulation, n=6. Data are shown as mean ± SD. (*) $p < 0.05$, (**) $p < 0.01$, (ns) not significant.
