# Supplementary material for: IL-1β priming triggers an adaptive stress response that enhances pancreatic β-cell resilience to subsequent cytotoxic inflammatory insult
Source: Cell Death Dis. 2025 Oct 21;16(1):744. doi: 10.1038/s41419-025-08059-0 (PMC12540857; doi:10.1038/s41419-025-08059-0)

iNOS – figure 1F

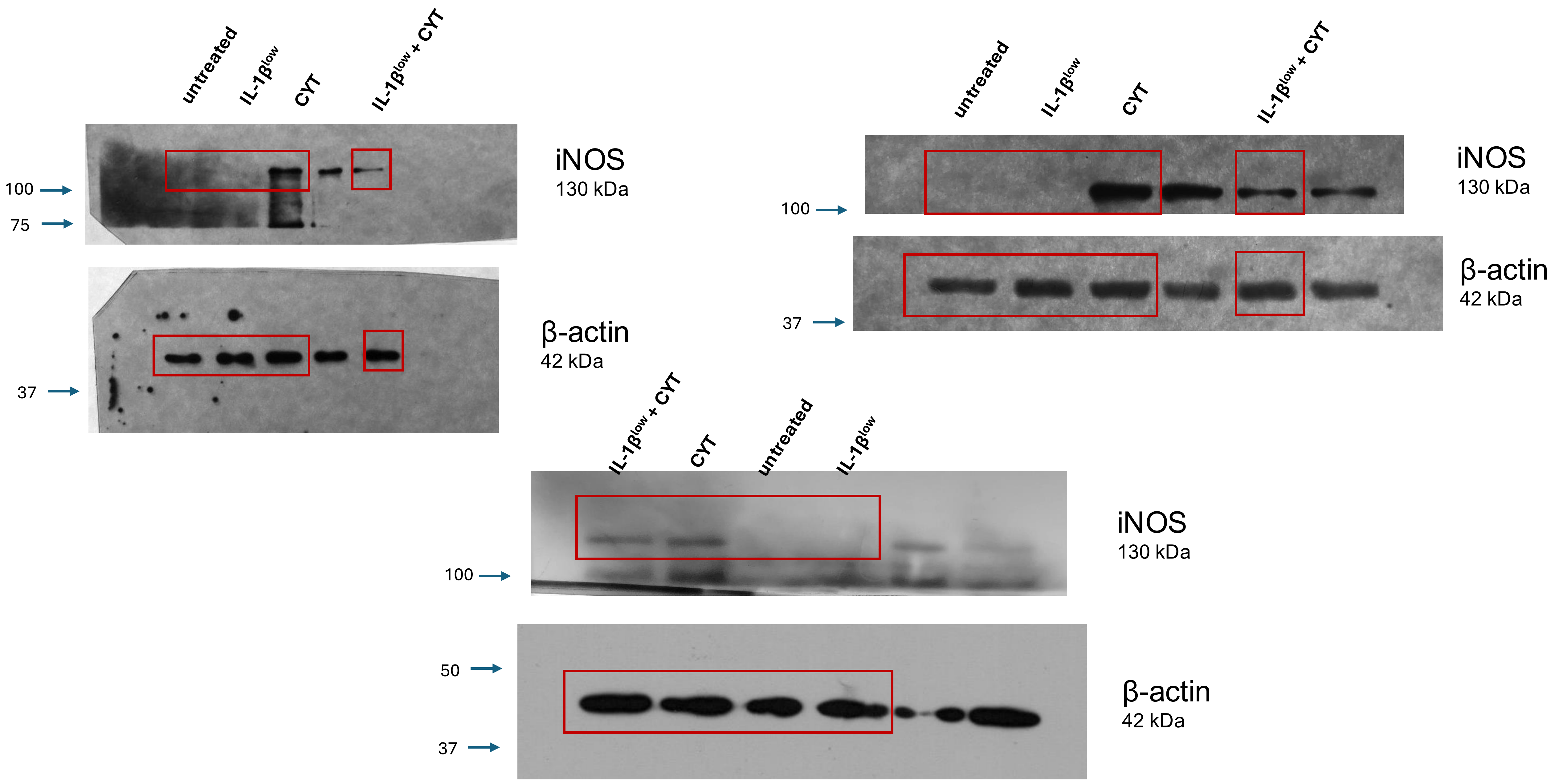

IκBα and p-IκBα - figure 2A

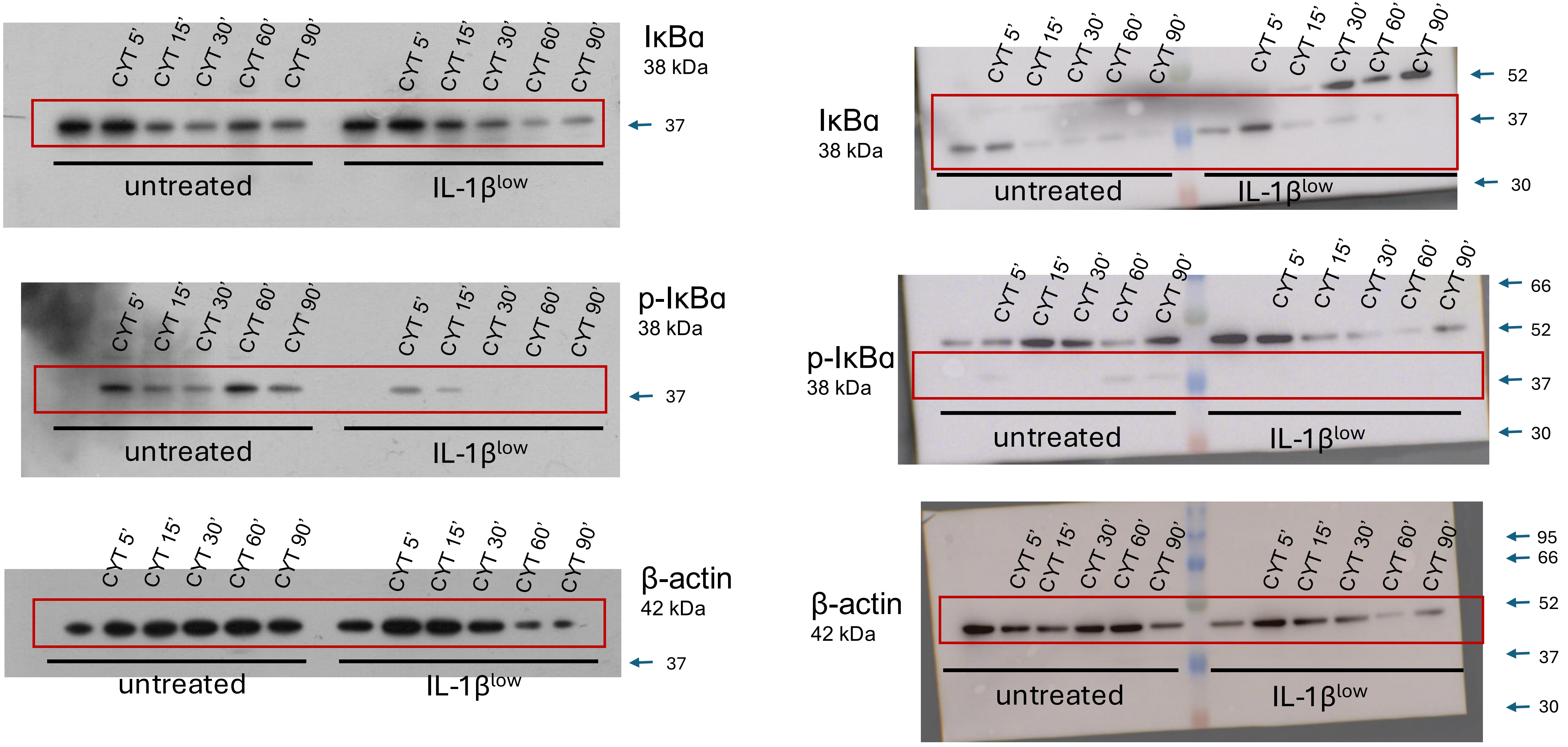

IκBα and p-IκBα - figure 2A

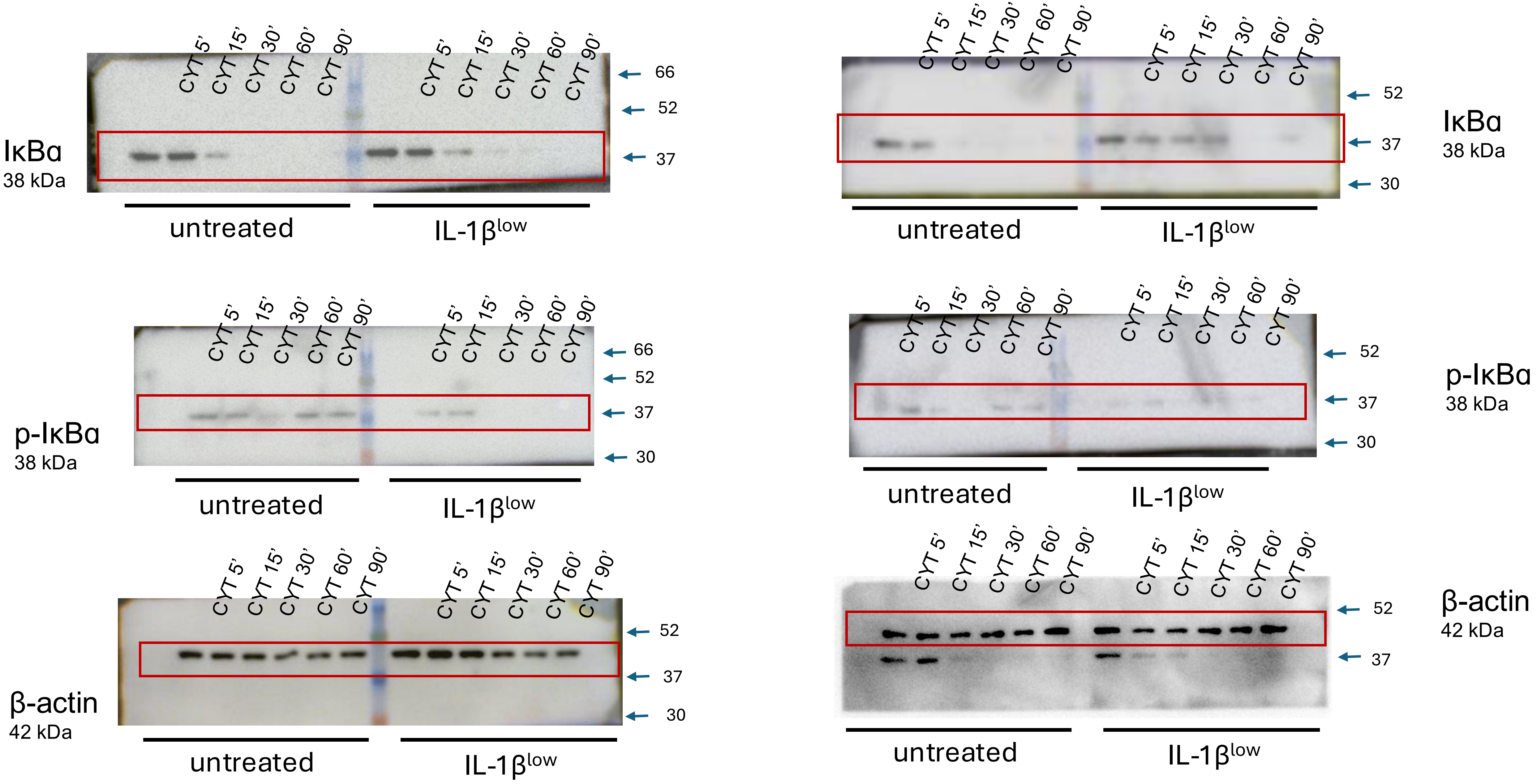

IκBα and p-IκBα - figure 2A

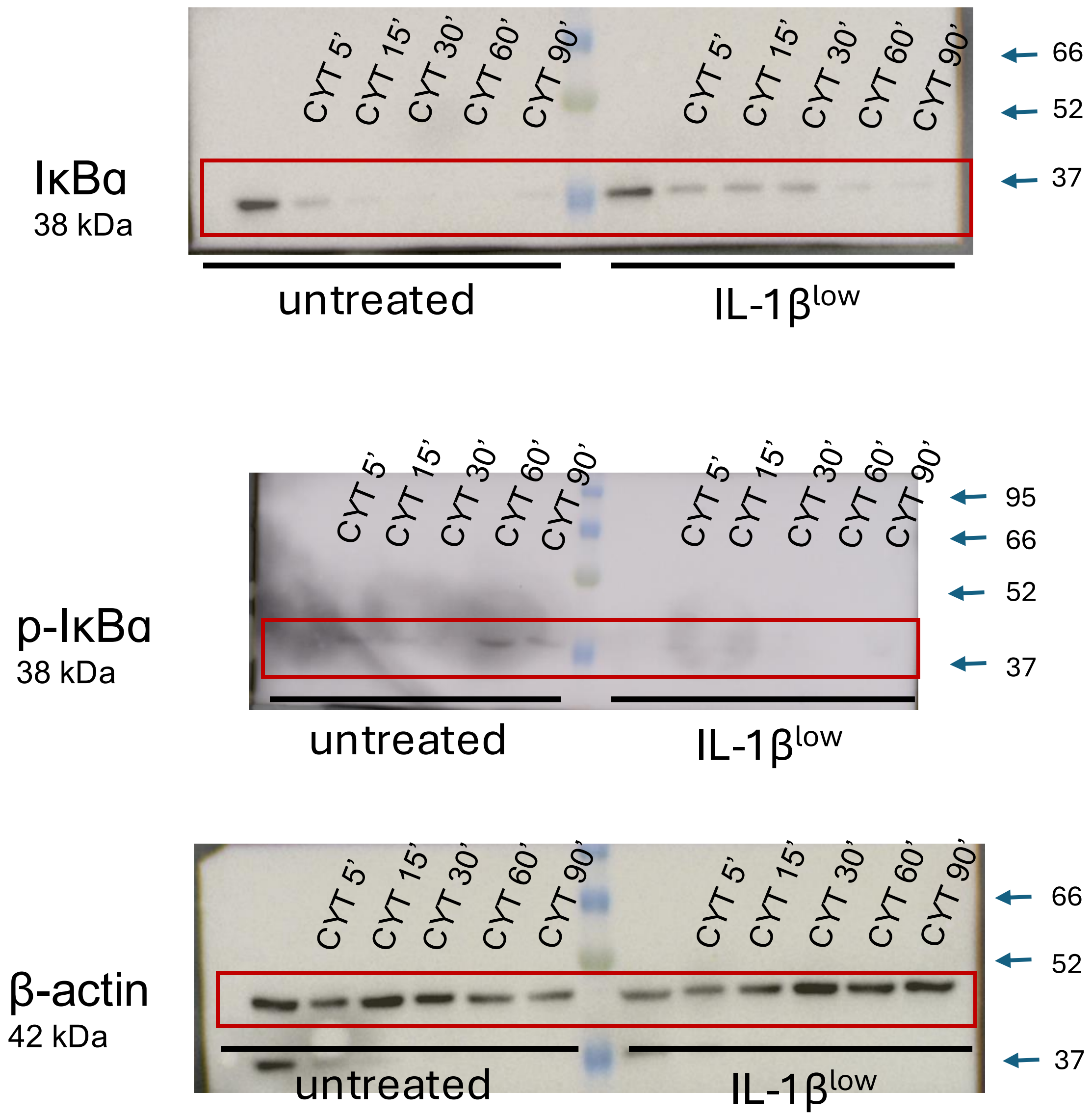

CHOP – figure 3H

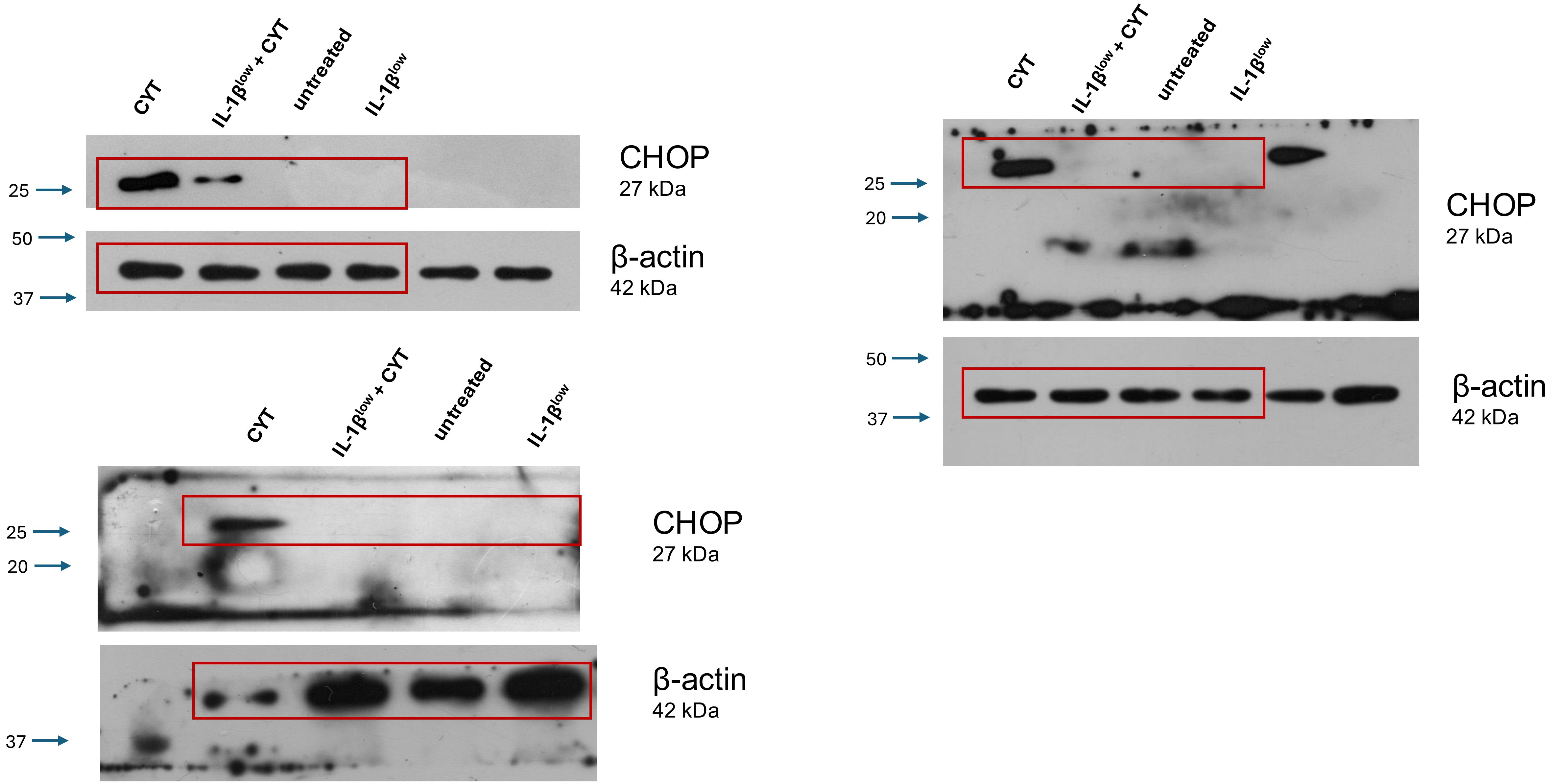

cleaved caspase-3 – figure 3H

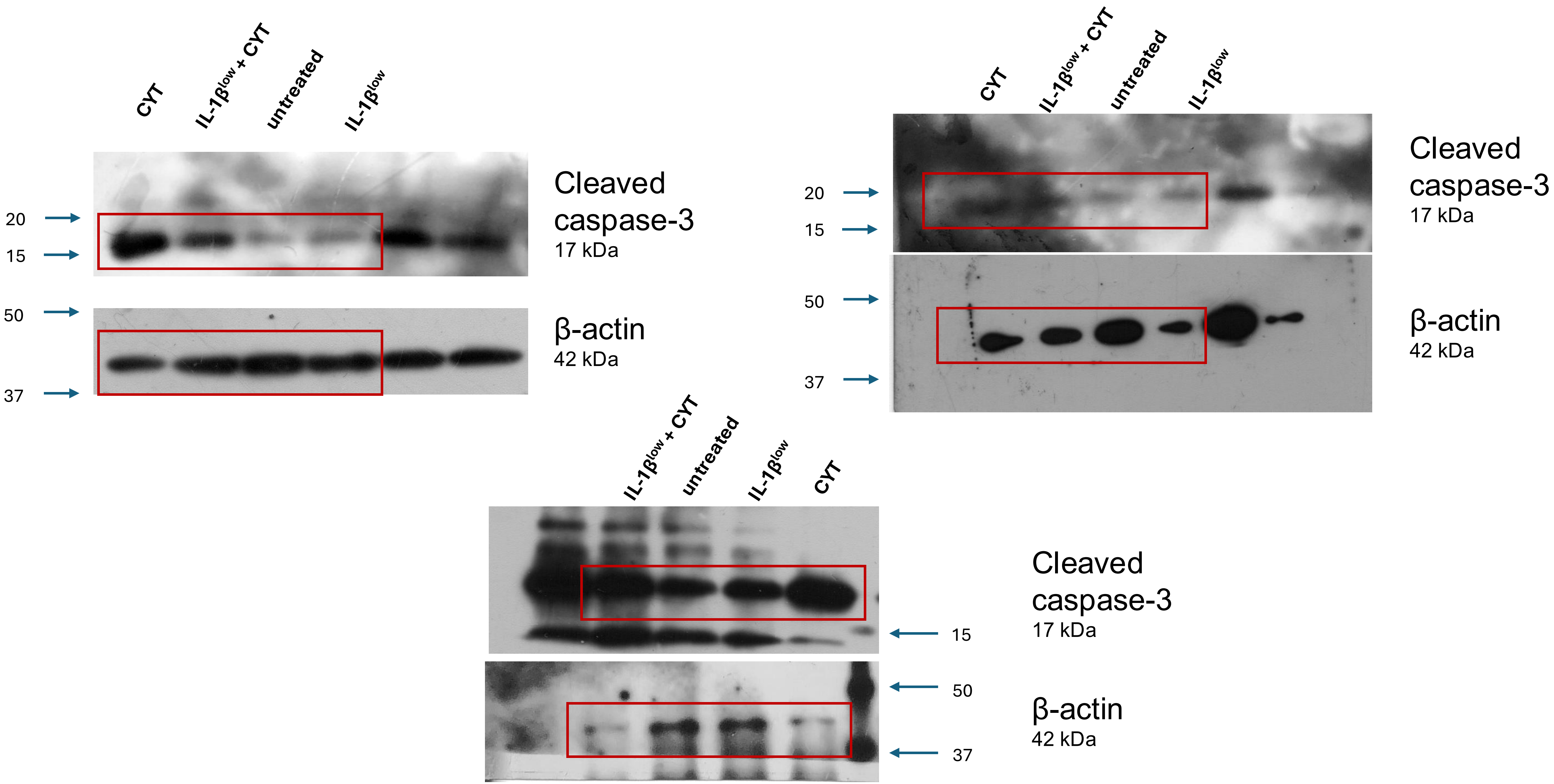

ATF4 - figure 4B

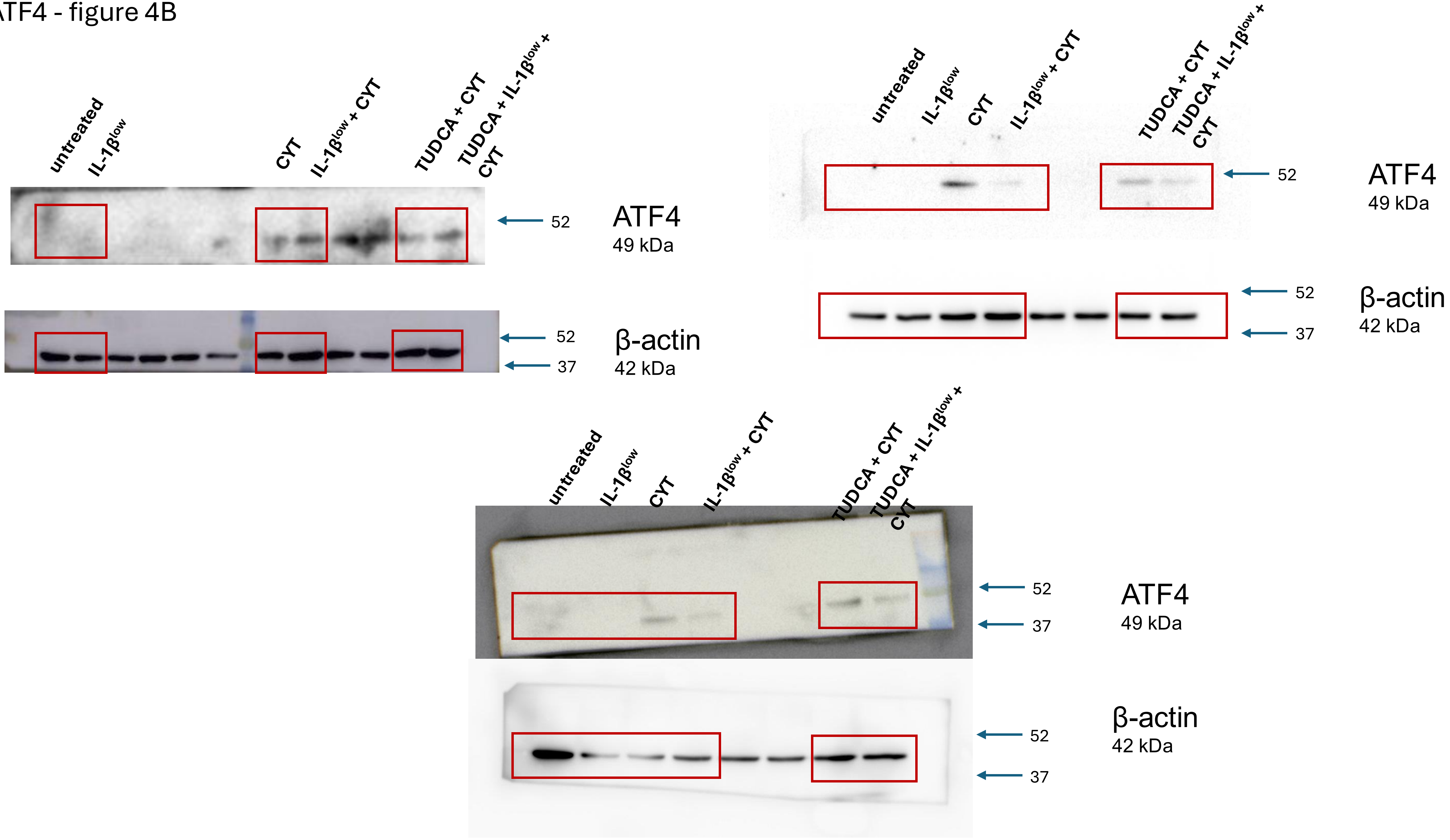

ATF4 - figure 4B

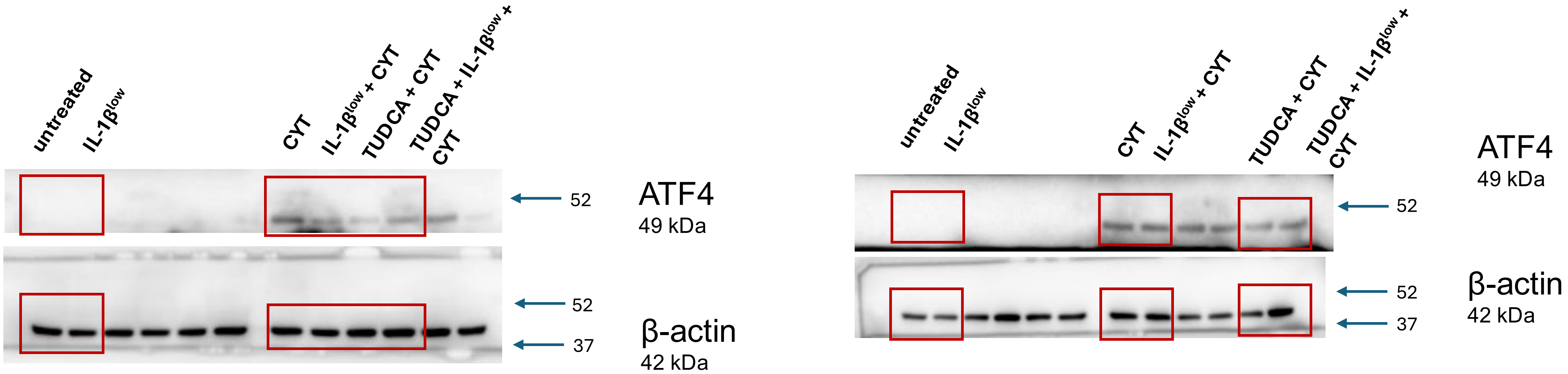

CHOP - figure 4B

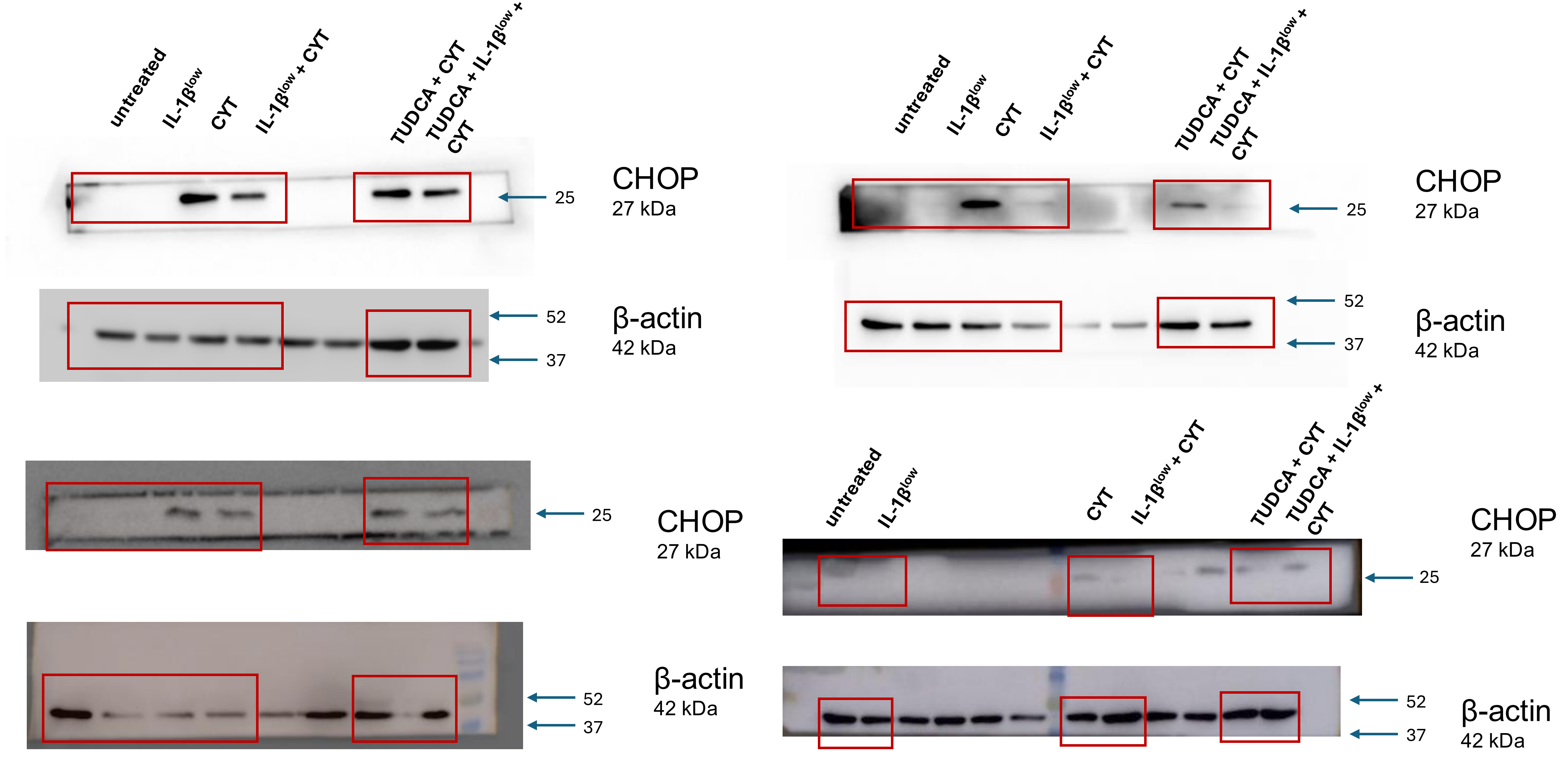

CHOP - figure 4B

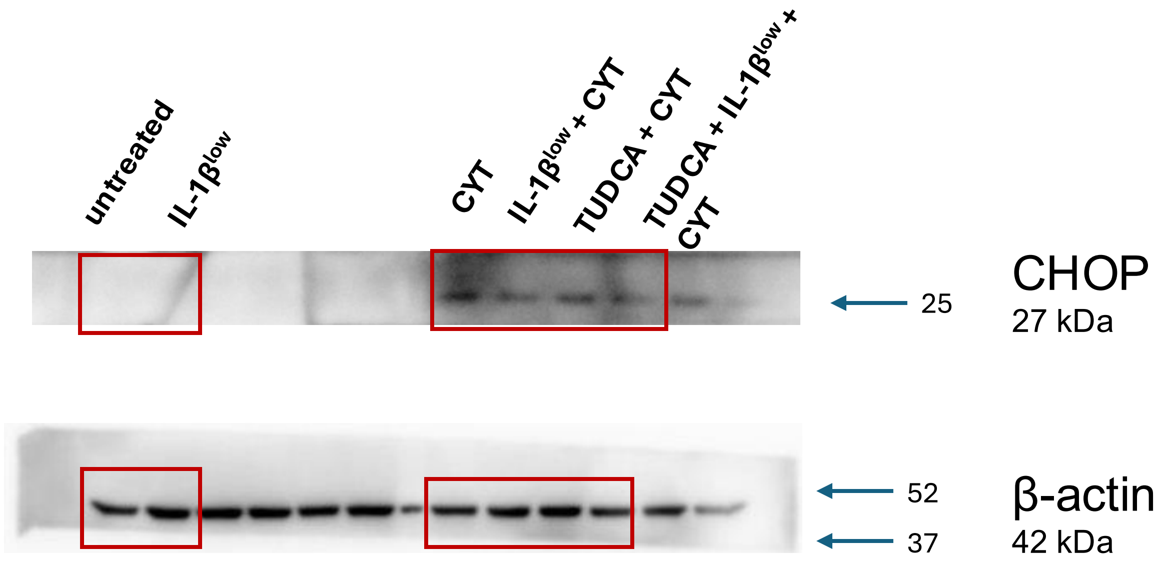

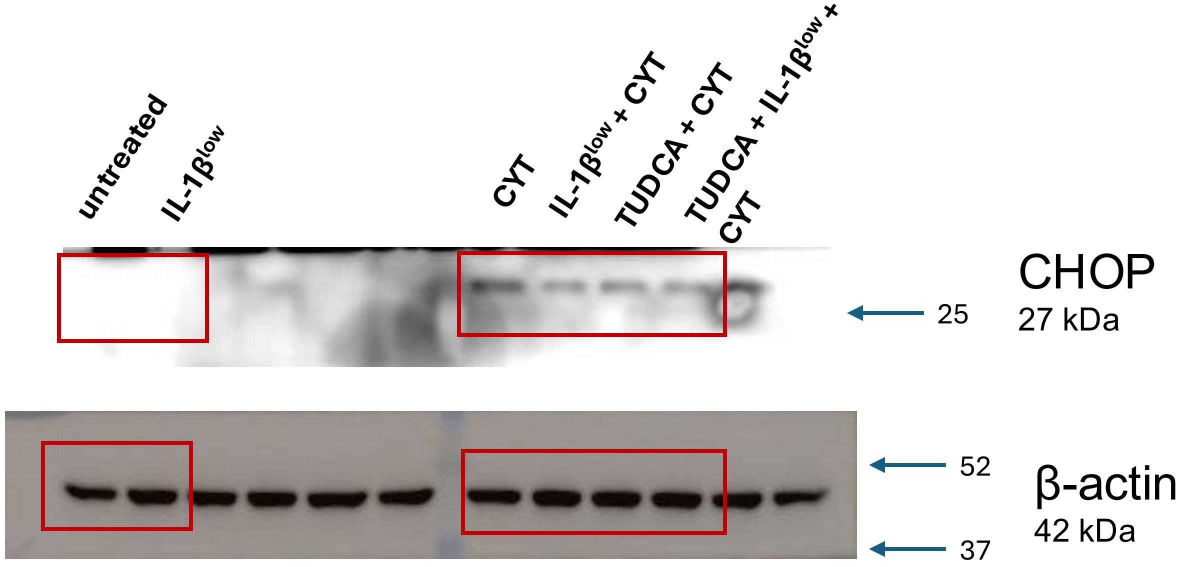

cleaved caspase-3 - figure 4B

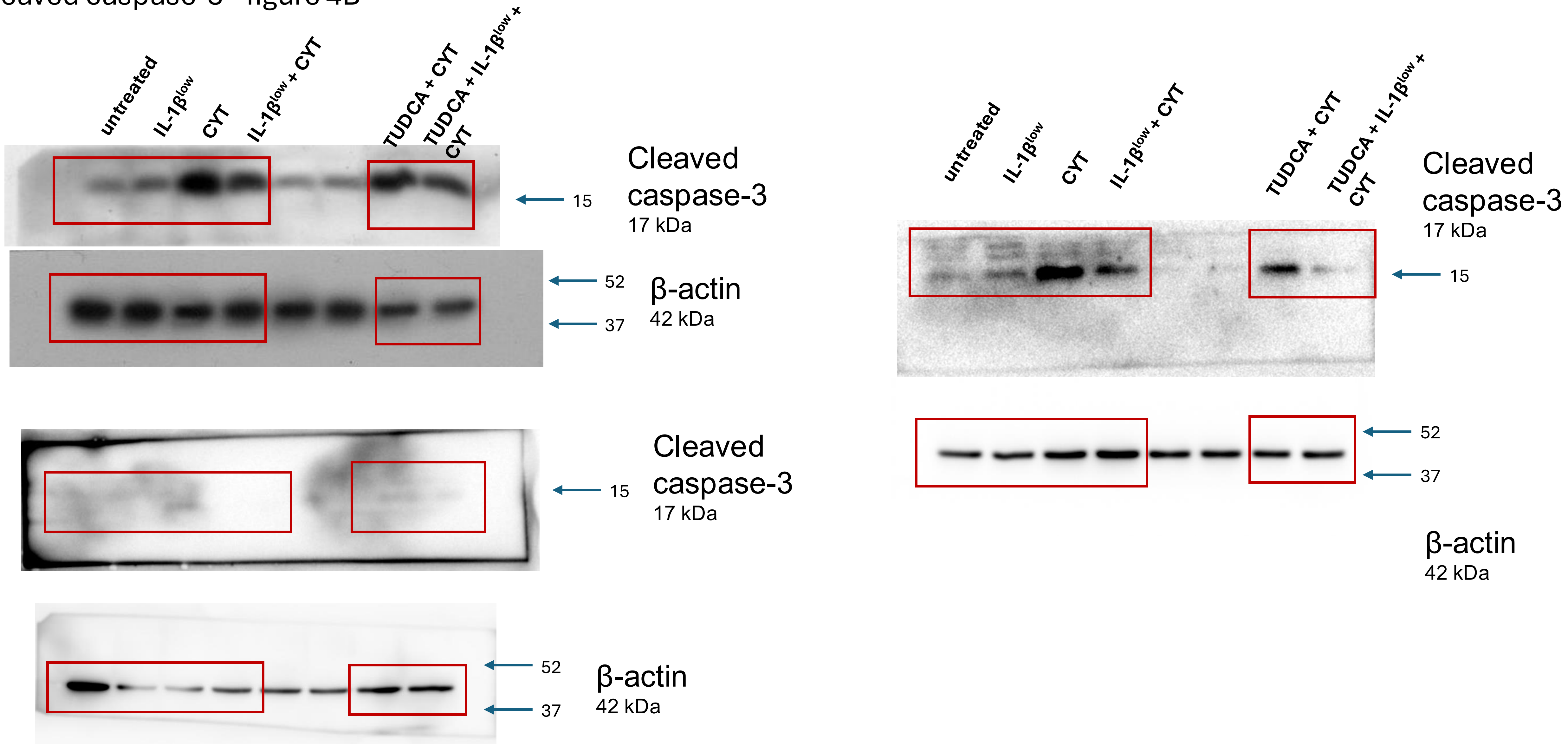

cleaved caspase-3 - figure 4B

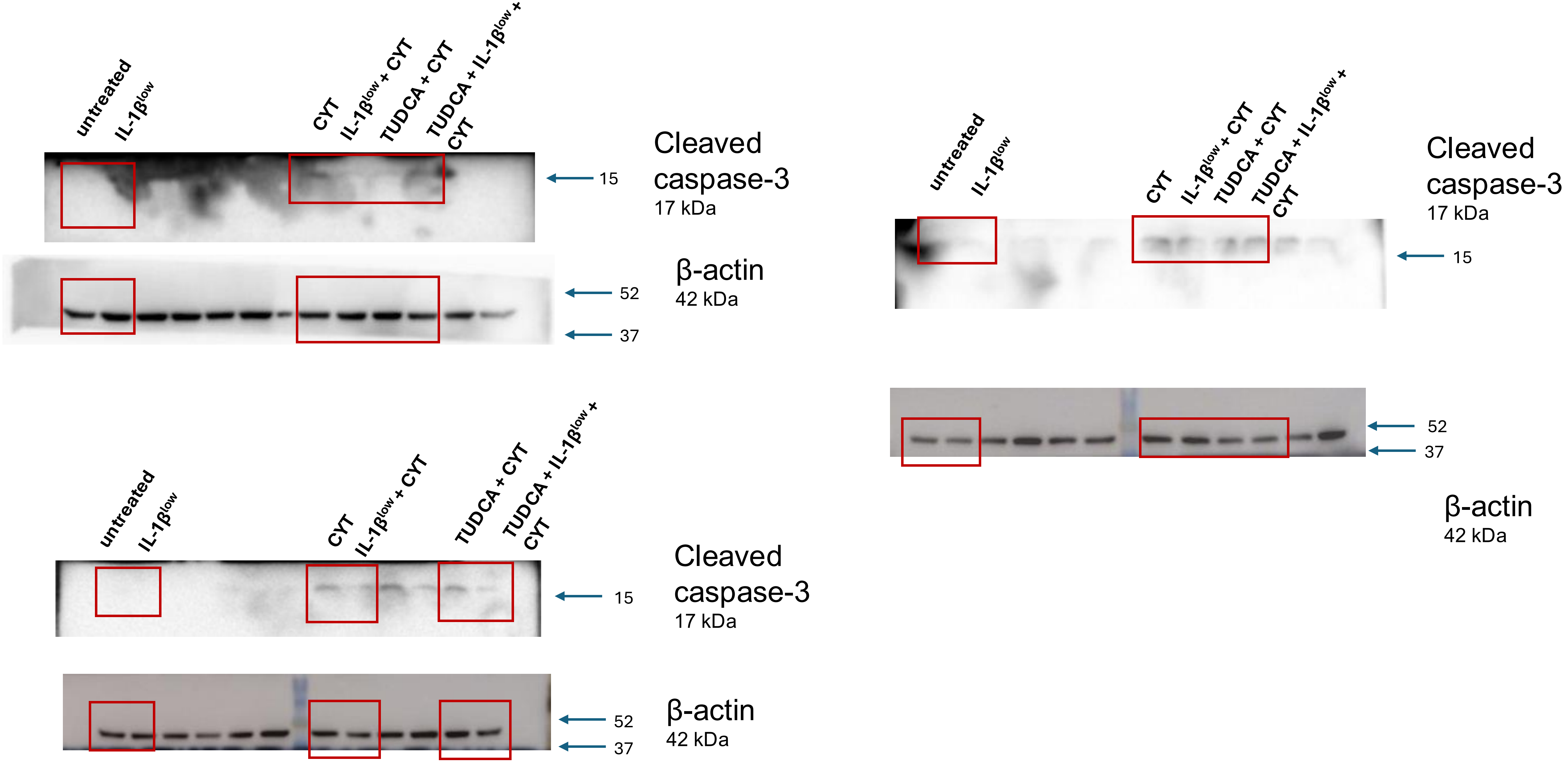

eIF2α and p-eIF2α - figure 5A

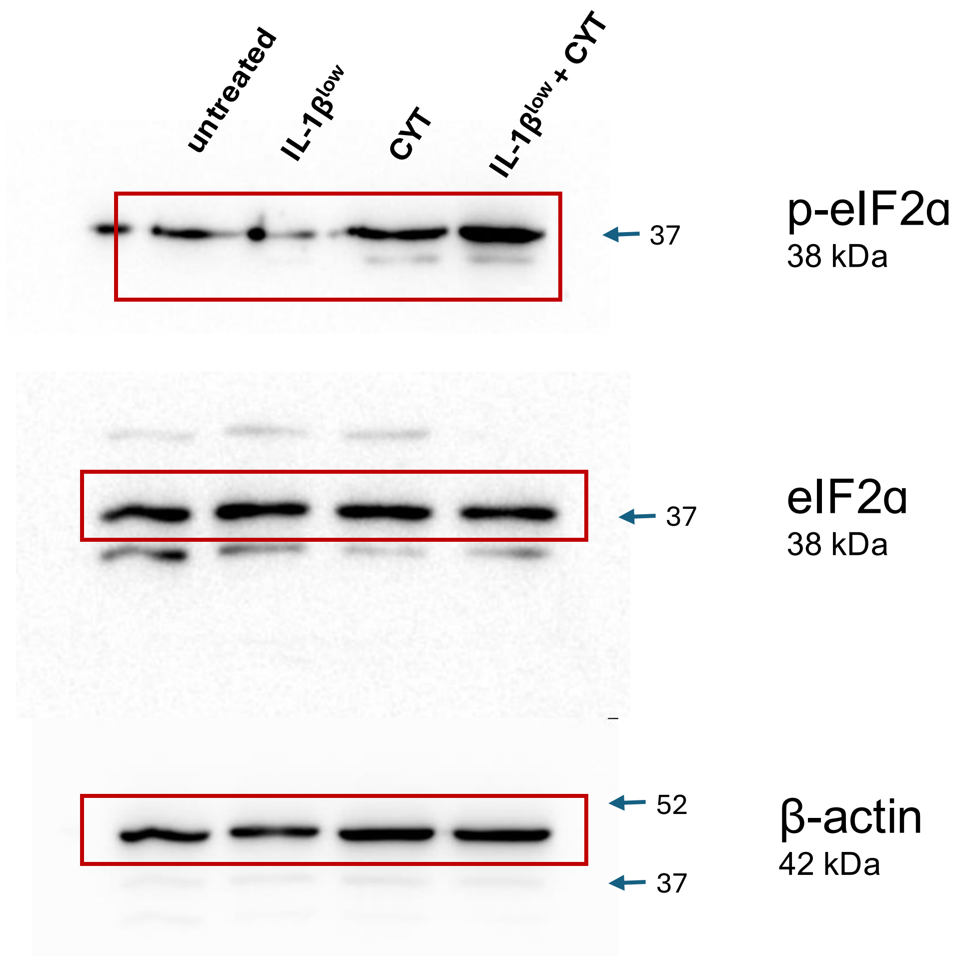

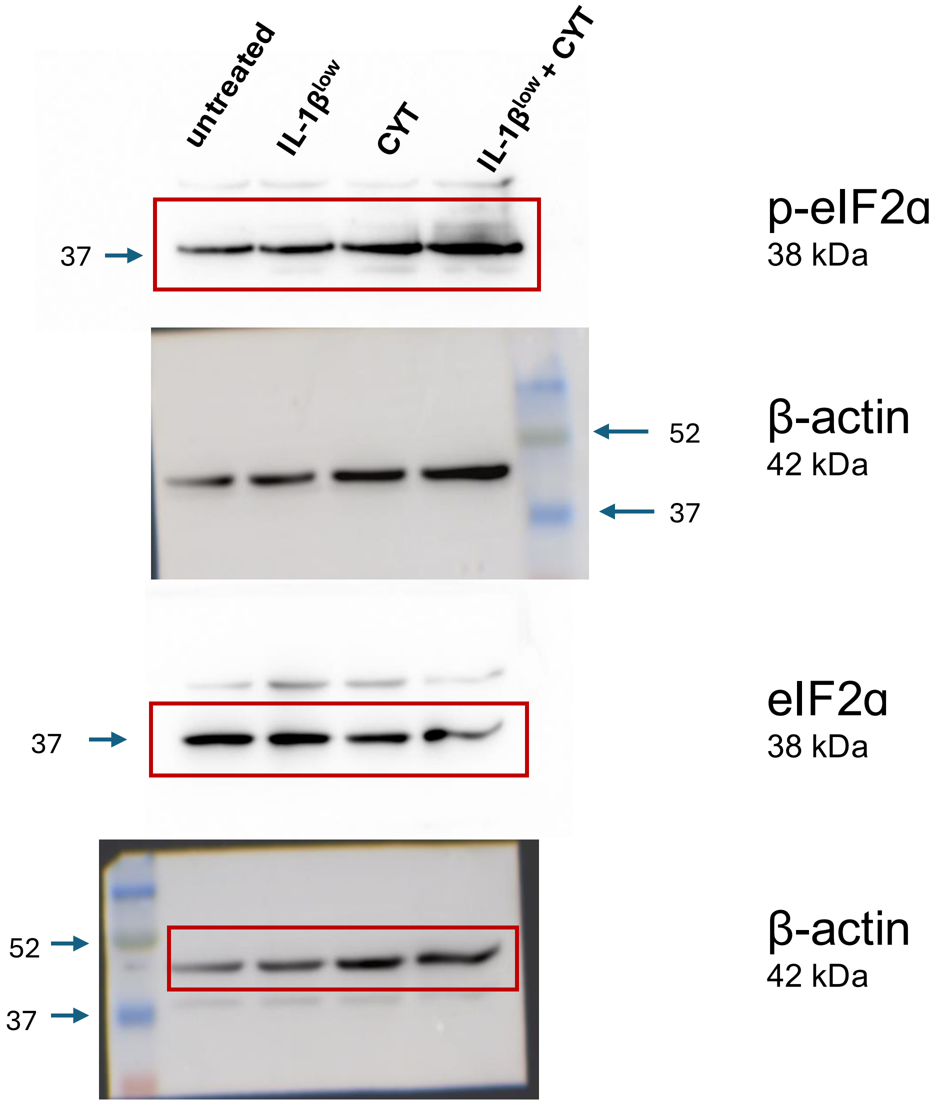

eIF2α and p-eIF2α - figure 5A

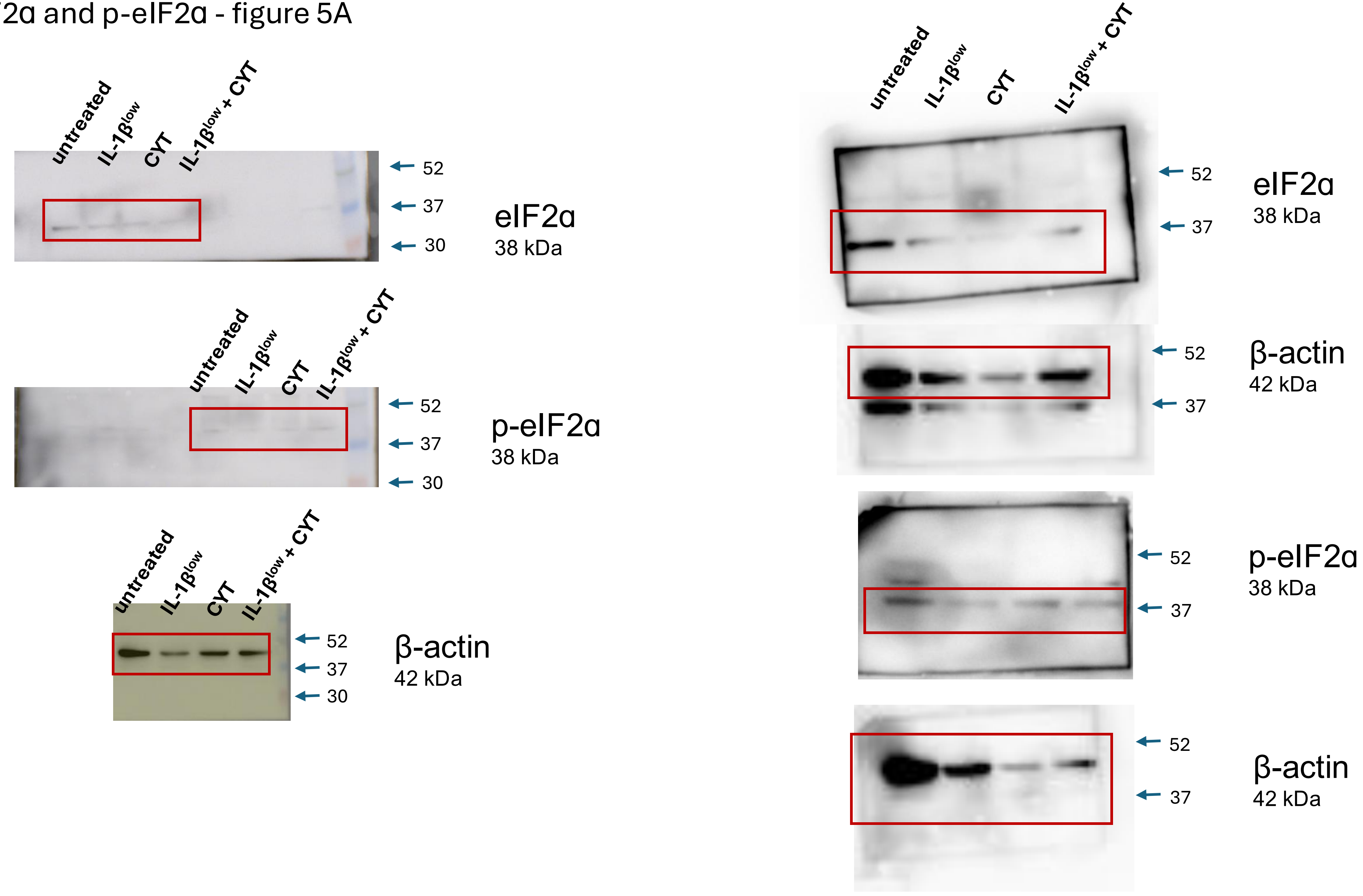

BiP - figure 5C

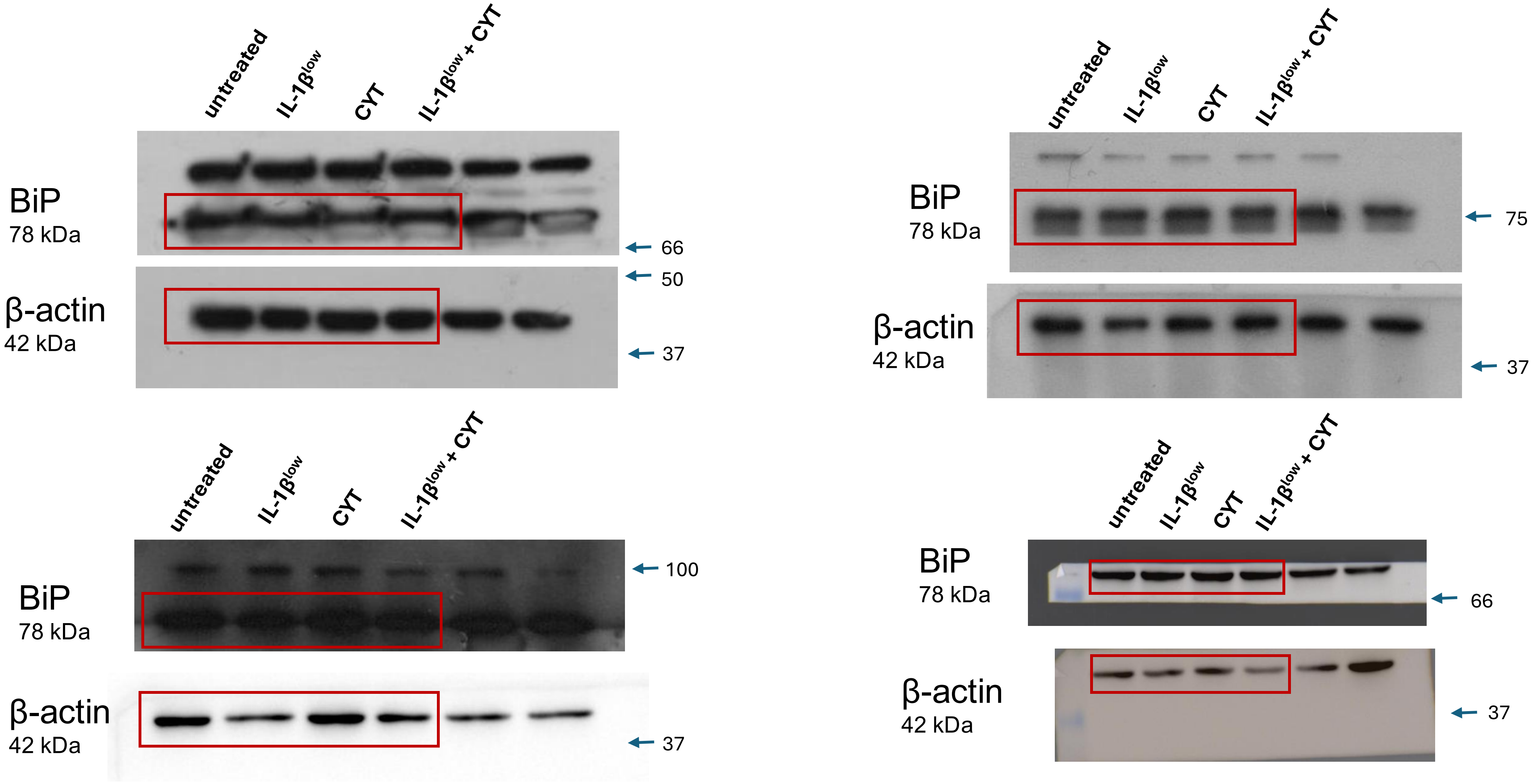

BiP - figure 5C

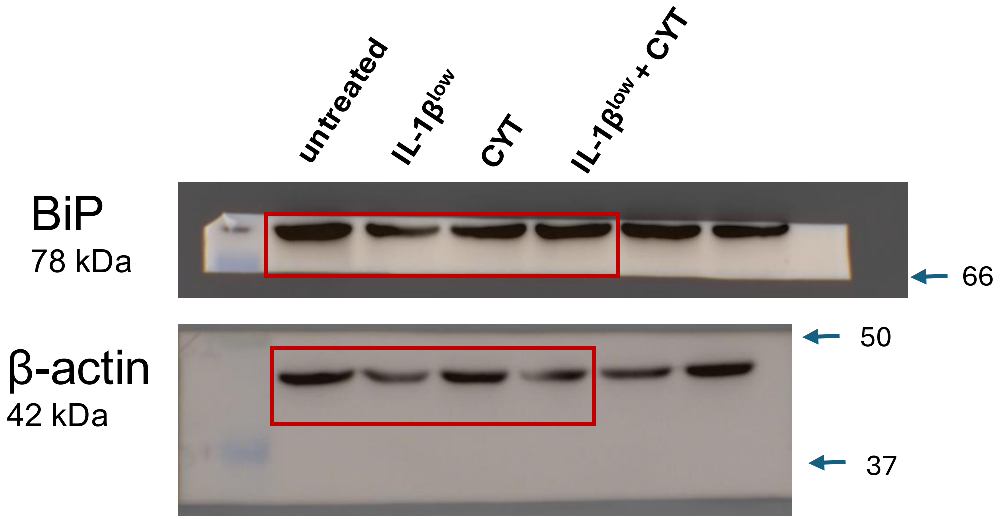

GRP94 - figure 5C

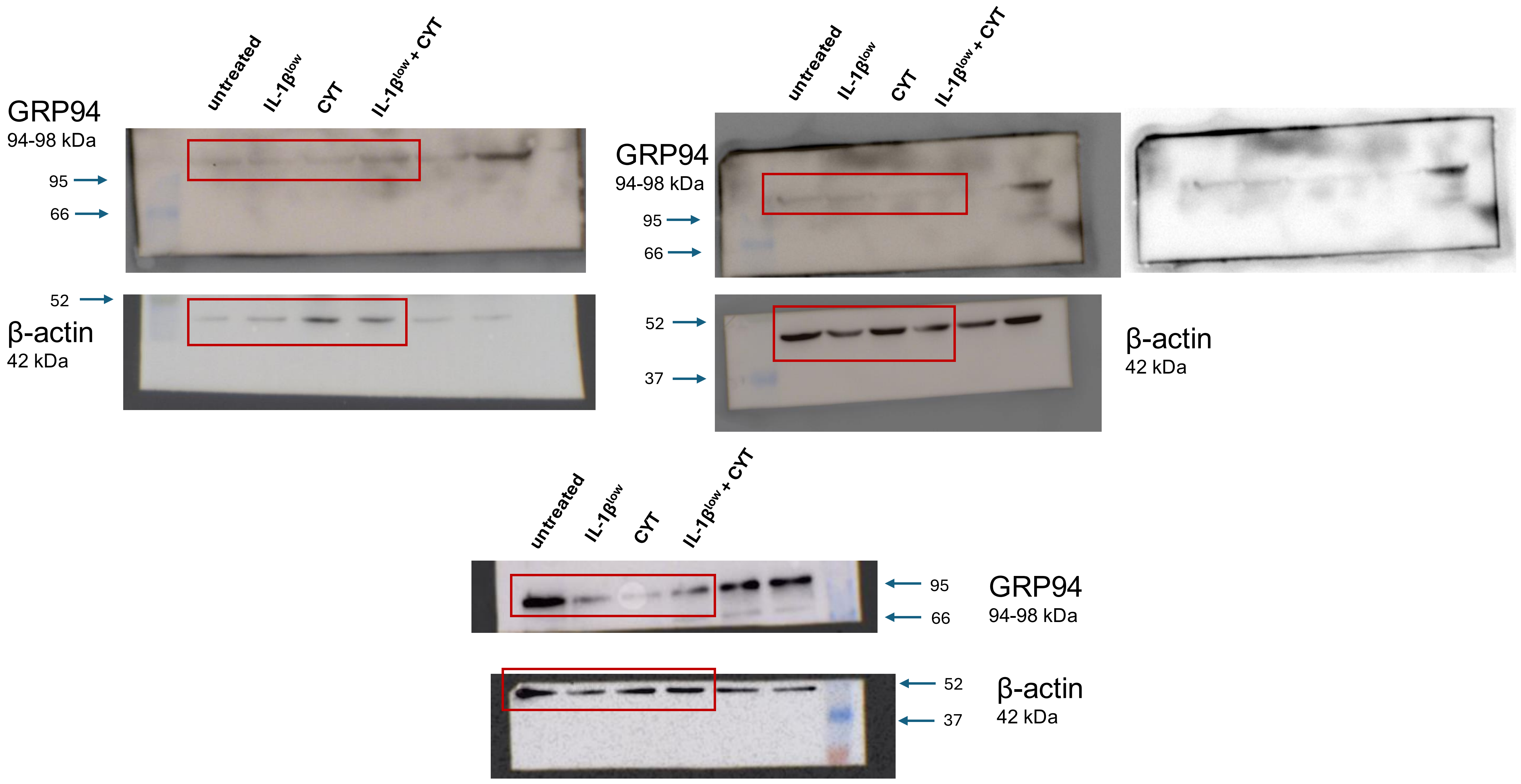

ORP150 - figure 5C

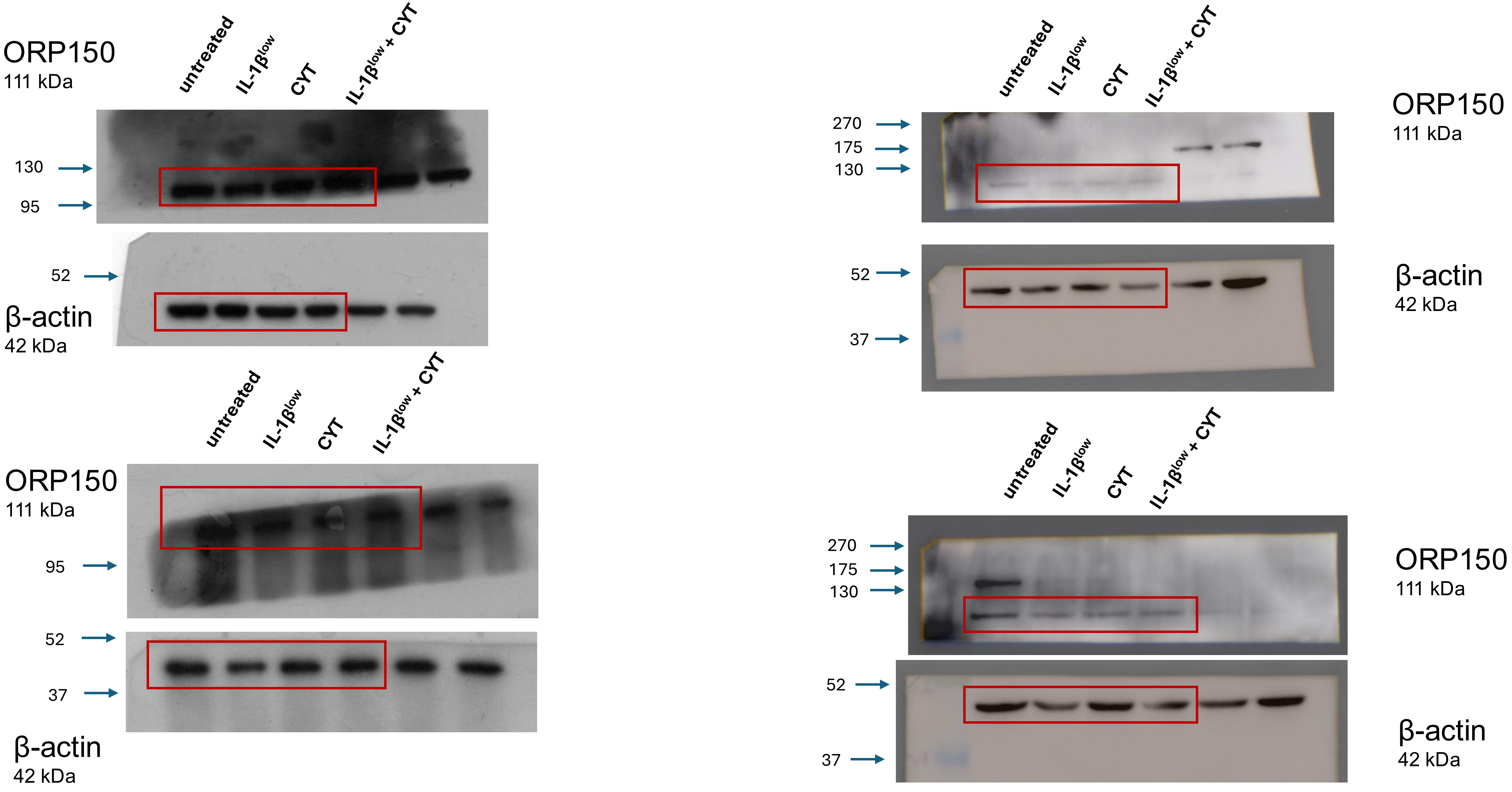

PDI - figure 5C

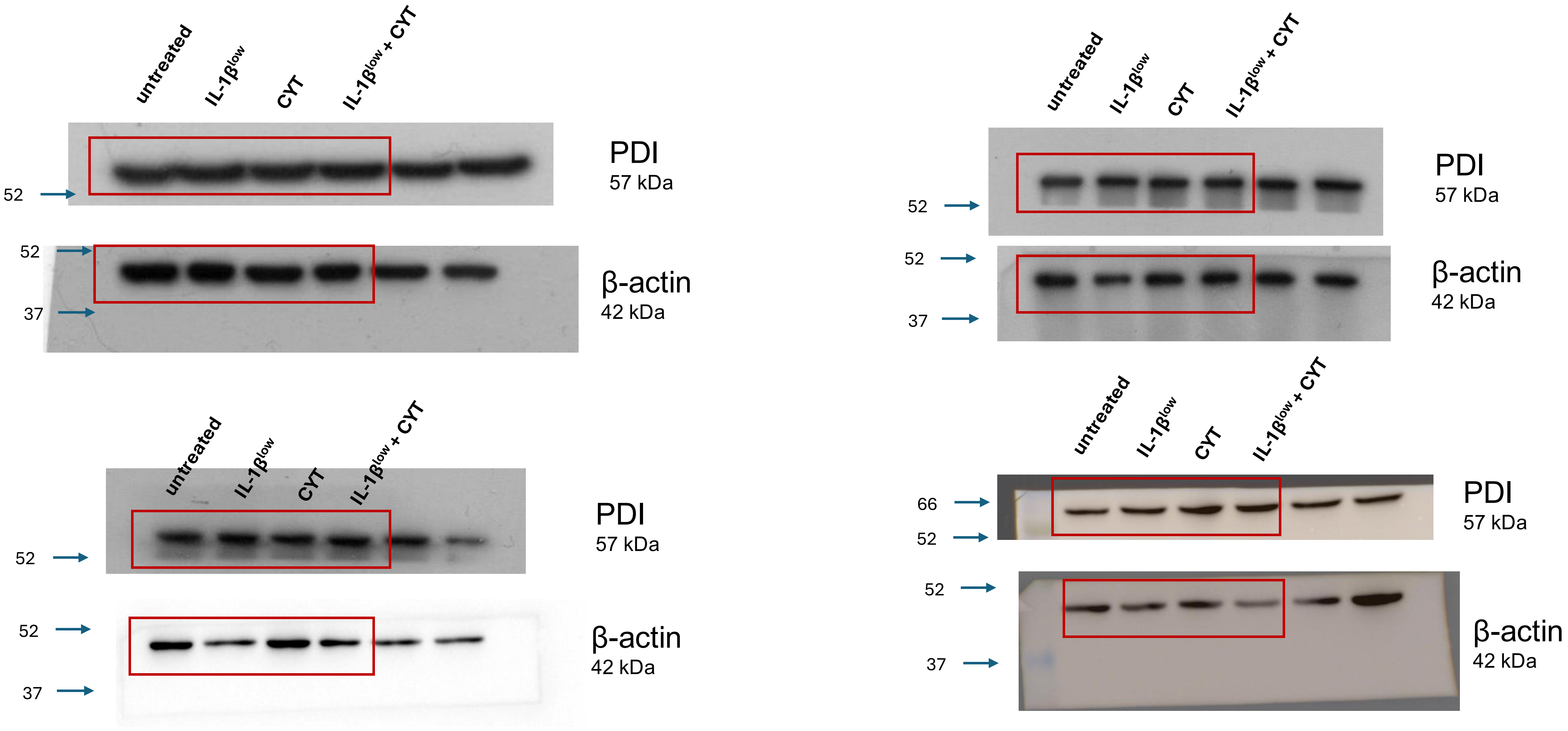

PDI - figure 5C

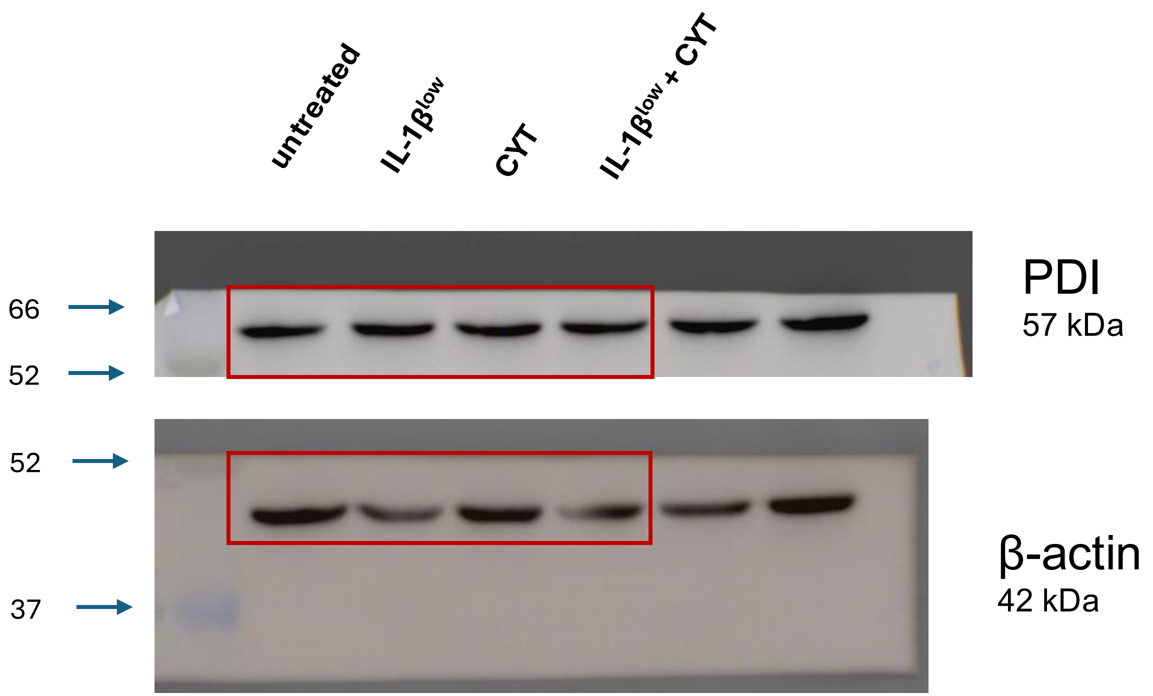

Supplement: Supplementary file 2 — uncropped original western blots [file 41419_2025_8059_MOESM2_ESM.pdf]
